# [18F]PI-2620 Tau PET signal across the aging and Alzheimer’s disease clinical spectrum

**DOI:** 10.1162/imag_a_00329

**Published:** 2024-10-24

**Authors:** Christina B. Young, Hillary Vossler, America Romero, Viktorija Smith, Jennifer Park, Alexandra N. Trelle, Joseph R. Winer, Edward N. Wilson, Michael M. Zeineh, Sharon J. Sha, Mehdi Khalighi, Maya V. Yutsis, Aimara P. Morales, David Anders, Greg Zaharchuk, Victor W. Henderson, Katrin I. Andreasson, Anthony D. Wagner, Kathleen L. Poston, Guido A. Davidzon, Elizabeth C. Mormino

**Affiliations:** Department of Neurology and Neurological Sciences, Stanford University School of Medicine, Palo Alto, CA, United States; Department of Radiology, Stanford University School of Medicine, Palo Alto, CA, United States; Wu Tsai Neurosciences Institute, Stanford University, Stanford, CA, United States; Department of Epidemiology and Population Health, Stanford University School of Medicine, Palo Alto, CA, United States; Department of Psychology, Stanford University, Stanford, CA, United States

**Keywords:** [18F]PI-2620, tau PET, Alzheimer’s disease, aging

## Abstract

[18F]PI-2620 is a second generation tracer that has shown high binding affinity for tau aggregation in Alzheimer’s disease (AD). However, [18F]PI-2620 signal in a large sample spanning the healthy aging and AD clinical spectrum as well as the stability of signal across different acquisition time windows has not yet been examined. Here, amyloid negative (Aβ-) cognitively unimpaired (CU; n = 49), amyloid positive (Aβ+) CU (n = 37), CU individuals with unknown amyloid status (n = 5), mild cognitive impairment (MCI; n = 14), dementia due to AD (n = 19), and non-AD neurodegenerative disorder (n = 54) participants were scanned with [18F]PI-2620 using a 45–75 min and/or 60–90 min acquisition time window. The impact of acquisition time on standardized uptake value ratio (SUVR) magnitude was first quantified with linear mixed models, and in participants and regions with high [18F]PI-2620 signal, SUVRs increased linearly up to 0.04 SUVR with each additional 5 min past injection time. We then accounted for differences in acquisition time using a voxel-wise correction approach and showed high correlations (all*r’*s ≥ 0.986) between SUVRs calculated from 45–75 min data and SUVRs from 60–90 min data that were interpolated to the 45–75 min scale in 15 participants who were scanned across both time windows. Using real and interpolated 45–75 min data, we next examined [18F]PI-2620 signal in Braak regions of interest and an off-target binding region (putamen) in Aβ- and Aβ+ CU, Aβ+ MCI, and Aβ+ AD dementia (n = 115) and showed that SUVRs in all Braak regions increased with greater disease severity. Within CU, higher Braak I SUVR was significantly associated with greater CSF pTau-181 (n = 35), and higher SUVRs were significantly associated with worse memory and language (n = 57). Thus, voxel-wise acquisition time corrections can be applied to combine [18F]PI-2620 datasets collected at different post-injection times, and once acquisition time is accounted for, [18F]PI-2620 signal shows the expected increases across the AD spectrum and can be used for detection of early tau elevations.

## 1 Introduction

Extracellular amyloid-beta (Aβ) plaques and intracellular neurofibrillary tangles comprising hyperphosphorylated tau are the two neuropathological hallmarks of Alzheimer’s disease (AD). Most existing studies have examined tau PET uptake across the AD spectrum using flortaucipir (FTP), but second-generation tracers, including [18F]PI-2620, have also shown high binding affinity for tau aggregation. The first human [18F]PI-2620 studies of AD were published in 2020 and have highlighted the ability of [18F]PI-2620 to detect 3R/4R tau. However, sample sizes have mostly been small and studies have used various acquisition times for collection of PET data (Table 1). This variability in post-injection time is important to consider given that the current timing recommendation is 45–75 min post-injection and that variability in acquisition protocols will likely increase when [18F]PI-2620 moves to the clinical real-world context and may vary longitudinally.

**Table 1.** Previously published [18F]PI-2620 studies ordered by acquisition time and increasing total sample size.

Study	Acquisition time	Study sample
( Beyer et al., 2020 )	0–60 min	N = 26 (7 AD with unknown Aβ status, 19 non-AD with unknown Aβ status)
( Messerschmidt et al., 2022 )	0–60 min	N = 46 (10 CU with unknown Aβ status, 36 non-AD with unknown Aβ status)
( Song, Beyer, et al., 2021 )	0–60 min	N = 50 (11 CU with unknown Aβ status, 10 Aβ+ AD dementia, 29 non-AD with unknown Aβ status)
( Palleis et al., 2021 )	0–60 min	N = 59 (14 Aβ- CU, 34 Aβ- non-AD, 11 Aβ+ non-AD)
( Rullmann et al., 2022 )	0–60 min	N = 64 (26 CU with unknown Aβ status, 38 Aβ+ AD dementia)
( Song, Scheifele, et al., 2021 )	0–60 min	N = 68 (10 CU with unknown Aβ status, 11 Aβ+ AD dementia, 47 non-AD with unknown Aβ status)
( Völter et al., 2023 )	0–60 min	N = 79 (15 Aβ- CU, 5 Aβ+ CU, 10 Aβ+ MCI, 6 Aβ+ AD dementia, 43 non-AD with unknown Aβ status)
( Brendel et al., 2020 )	0–60 min	N = 90 (10 CU with unknown Aβ status, 2 Aβ+ MCI, 8 Aβ+ AD dementia, 70 non-AD with unknown Aβ status)
( Katzdobler et al., 2023 )	0–60 min	N = 206 (12 Aβ- CU, 47 Aβ+ AD, 9 AD with unknown Aβ status, 15 Aβ- non-AD, 123 non-AD with unknown Aβ status)
( Bun et al., 2022 )	60–90 min	N = 14 (7 Aβ- CU, 7 Aβ+ AD dementia)
( Tezuka et al., 2021 )	60–90 min	N = 21 (7 Aβ- CU, 8 Aβ+ AD dementia, 2 Aβ- non-AD, 3 Aβ+ non-AD, 1 non-AD with unknown Aβ status)
( Oh et al., 2020 )	60–90 min	N = 26 (3 Aβ- CU, 3 Aβ+ MCI, 6 Aβ+ AD dementia, 13 Aβ- non-AD, 1 Aβ+ non-AD)
( Chen et al., 2023 )	60–90 min	N = 43 (21 Aβ- CU, 7 Aβ+ CU, 2 CU with unknown Aβ status, 1 Aβ- MCI, 3 Aβ+ MCI, 4 Aβ+ AD dementia, 1 AD dementia with unknown Aβ status, 4 non-AD with unknown Aβ status)
( Bullich et al., 2022 )	60–90 min	N = 74 (72 Aβ+ MCI, 2 Aβ+ AD dementia)
( Mormino et al., 2021 )	0–90 min (with a break from 30–50 min) or 60–90 min	N = 49 (36 CU with unknown Aβ status, 7 Aβ+ MCI/AD dementia, 6 non-AD with unknown Aβ status)
( Mueller et al., 2020 )	0–180 min (with a break from 90–120 min)	N = 22 (10 Aβ- CU, 12 Aβ+ AD dementia)
( Blazhenets et al., 2023 )	30–60 min	N = 81 (23 Aβ- CU, 16 Aβ+ MCI/AD dementia, 5 Aβ- MCI/dementia, 8 Aβ+ non-AD, 24 Aβ- non-AD, 5 non-AD with unknown Aβ status)
( Jantarato et al., 2021 )	30–75 min	N = 69 (26 CU with unknown Aβ status, 36 MCI with unknown Aβ status, 7 AD with unknown Aβ status)
( Thanapornsangsuth et al., 2023 )	unknown	N = 51 (4 Aβ- CU, 10 Aβ- MCI, 22 Aβ+ MCI, 2 Aβ- dementia, 13 Aβ+ dementia; 27/51 of their sample had a clinical diagnosis of AD and 24/51 participants had a clinical diagnosis of non-AD)

Our first aim was to examine the temporal stability of [18F]PI-2620 standardized uptake value ratios (SUVRs) in order to combine data from different acquisition time protocols and understand results across studies. Our second aim was to characterize the patterns of tau pathology identified with [18F]PI-2620 in a single large AD clinical spectrum sample to confirm expected associations with disease severity and cognitive impairment.

## 2 Methods

### 2.1 Participants

In total, 178 participants underwent a [18F]PI-2620 tau PET-MR scan (Table 2;Supplementary Fig. S1). Amyloid status was determined by plasma, CSF, or amyloid PET. Participants were recruited from the Stanford Alzheimer’s Disease Research Center (ADRC), the Stanford Aging and Memory Study (SAMS), and the Stanford Center for Memory Disorders. All participants provided written informed consent or assent, and protocols were approved by the Stanford Institutional Review Board.

**Table 2.** Demographics of the (A) full dataset, (B) subset of cognitively unimpaired (CU) participants with available CSF pTau-181, and (C) subset of Stanford Alzheimer’s Disease Research Center (ADRC) participants on the aging and AD clinical spectrum with available cognitive data.

**(A) Full dataset**					
	**CU (n** **=** **91)**	**MCI (n** **=** **14)**	**AD Dementia (n** **=** **19)**	**Non-AD (n** **=** **54)**	**Overall (n** **=** **178)**
**Age**					
Mean (SD)	72.9 (7.8)	71.3 (11.3)	72.7 (8.7)	74.2 (7.9)	73.2 (8.2)
Median [Min, Max]	73.1 [41.5, 91.5]	69.7 [47.3, 89.9]	75.0 [57.8, 85.7]	74.6 [53.7, 94.3]	73.5 [41.5, 94.3]
**Sex**					
Female	55 (60.4%)	5 (35.7%)	12 (63.2%)	17 (31.5%)	89 (50.0%)
Male	36 (39.6%)	9 (64.3%)	7 (36.8%)	37 (68.5%)	89 (50.0%)
**Amyloid Status**					
Aβ-	49 (60.4%)	2 (14.3%)	0 (0%)	24 (44.4%)	75 (42.1%)
Aβ+	37 (40.7%)	12 (85.7%)	17 (89.5%)	24 (44.4%)	90 (50.6%)
Unknown	5 (5.5%)	0 (0%)	2 (10.5%)	6 (11.1%)	13 (7.3%)

**(B) Subset of CU participants with CSF pTau-181**					
	**Aβ- CU (n** **=** **26)**		**Aβ+ CU (n** **=** **9)**		**Overall (n** **=** **35)**
**Age**					
Mean (SD)	68.9 (4.8)		72.9 (5.0)		69.9 (5.1)
Median [Min, Max]	67.1 [61.7, 79.6]		74.4 [63.7, 78.2]		69.1 [61.7, 79.6]
**Sex**					
Female	12 (46.2%)		4 (44.4%)		16 (45.7%)
Male	14 (53.9%)		5 (55.6%)		19 (54.3%)
**Years between CSF and Tau PET**					
Mean (SD)	-0.7 (0.4)		-1.0 (0.5)		-0.6 (0.4)
Median [Min, Max]	-0.8 [-1.2, 0.0]		-1.0 [-1.8, -0.3]		-0.8 [-1.8, 0.0]
**CSF pTau-181**					
Mean (SD)	38.3 (11.0)		57.7 (19.9)		43.3 (16.0)
Median [Min, Max]	37.6 [12.7, 63.2]		55.5 [35.9, 91.0]		39.5 [12.7, 91.0]

**(C) Subset of ADRC aging and AD participants with cognitive data**					
	**Aβ- CU (n** **=** **16)**	**Aβ+ CU (n** **=** **21)**	**Aβ+ MCI (n** **=** **10)**	**Aβ+ AD Dementia (n** **=** **10)**	**Overall (n** **=** **57)**
**Age**					
Mean (SD)	72.8 (11.5)	74.6 (9.0)	71.8 (9.5)	76.1 (6.1)	73.9 (9.3)
Median [Min, Max]	73.6 (41.5, 86.7)	73.7 (54.6, 91.5)	69.7 (56.3, 89.9)	76.9 (62.3, 85.7)	74.0 (41.5, 91.5)
**Sex**					
Female	14 (87.5 %)	14 (66.7 %)	3 (30.0 %)	6 (60.0 %)	37 (64.9 %)
Male	2 (12.5 %)	7 (33.3 %)	7 (70.0 %)	4 (40.0 %)	20 (35.1 %)
**Education**					
Mean (SD)	17.4 (2.3)	18.1 (1.9)	16.6 (1.9)	17.5 (2.6)	17.5 (2.1)
Median [Min, Max]	18.0 (12.0, 20.0)	18.0 (14.0, 20.0)	16.0 (14.0, 20.0)	18.0 (14.0, 20.0)	18.0 (12.0, 20.0)
**Memory Score**					
Mean (SD)	0.3 (0.8)	-0.0 (0.9)	-2.1 (1.3)	-3.7 (0.3)	-0.8 (1.7)
Median [Min, Max]	0.4 (-1.2, 1.6)	0.2 (-2.2, 1.2)	-1.9 (-3.9, -0.5)	-3.7 (-4.1, -3.3)	-0.2 (-4.1, 1.6)
Missing	2 (12.5%)	2 (9.5%)	3 (30.0%)	3 (30.0%)	10 (17.5%)
**Executive Function Score**					
Mean (SD)	0.1 (0.4)	-0.0 (0.6)	-1.0 (0.9)	-1.6 (0.8)	-0.4 (0.9)
Median [Min, Max]	0.2 (-0.6, 0.9)	-0.0 (-1.6, 1.1)	-0.7 (-2.8, -0.2)	-2.1 (-2.4, -0.3)	-0.2 (-2.8, 1.1)
Missing	2 (12.5%)	4 (19.0%)	3 (30.0%)	3 (30.0%)	12 (21.1%)
**Language Score**					
Mean (SD)	-0.1 (0.4)	0.2 (0.8)	-0.9 (0.8)	-1.5 (1.2)	-0.3 (1.0)
Median [Min, Max]	0.1 (-0.7, 0.6)	0.3 (-1.3, 1.6)	-1.0 (-2.1, 0.0)	-0.9 (-3.4, -0.2)	-0.1 (-3.4, 1.6)
Missing	0 (0%)	0 (0%)	0 (0%)	1 (10.0%)	1 (1.8%)
**Visuospatial Score**					
Mean (SD)	-0.2 (0.4)	-0.0 (1.0)	0.2 (0.5)	-0.9 (2.2)	-0.1 (1.1)
Median [Min, Max]	-0.1 (-0.6, 0.9)	0.2 (-2.3, 1.0)	0.3 (-0.5, 0.7)	-0.1 (-5.6, 0.8)	0.2 (-5.6, 1.0)
Missing	2 (12.5%)	2 (9.5%)	3 (30.0%)	3 (30.0%)	10 (17.5%)

### 2.2 [18F]PI-2620 data

#### 2.2.1 Data acquisition

[18F]PI-2620 was synthesized using a previously published protocol (Bullich et al., 2020;Mormino et al., 2021;Mueller et al., 2020) and PET scanning was completed using a simultaneous time-of-flight (TOF)–enabled PET/MRI scanner (SIGNA 3T, GE HealthCare). Following a 5–10 mCi injection of [18F]PI-2620 into an antecubital vein, 49 participants had data collected 60–90 min post-injection, 114 had data collected 45–75 min post-injection, and 15 had data collected from 45–90 min post-injection encompassing both 45–75 min and 60–90 min time windows. Data were reconstructed into 5-min frames and frames were realigned and summed. SUVRs for each FreeSurfer v7 ROI were calculated using an inferior cerebellum reference region.

#### 2.2.2 Timing acquisition adjustment

We adjusted 60–90 min data to the 45–75 SUVR scale using methods described byPontecorvo et al. (2019). Briefly, a slope was first calculated for every voxel using all 5-min frame data. The summed 60–90 min data and the calculated slope were then used to interpolate the estimated value at 60 min (midpoint of 45–75 min), thereby adjusting the 60–90 min data to the 45–75 min scale.

#### 2.2.3 Regions of interest (ROIs) and tau status

We focused on 6 ROIs that mimic Braak stages (Biel et al., 2021) and the putamen, a region with off-target binding with FTP (Choi et al., 2018). We additionally examined [18F]PI-2620 signal across the brain using all aparc+aseg regions except ventricle, brainstem, corpus callosum, and cerebellum. Tau positive (T+) status was defined for each brain region as 2 standard deviations greater than the mean of Aβ- CUs (n = 49) using acquired 45–75 min and interpolated 45–75 min data.

### 2.3 Cognition

ADRC participants completed the National Alzheimer’s Coordinating Center Uniform Data Set 3 neuropsychological battery and Stanford-specific supplemental cognitive tests. Composite scores representing memory, executive functioning, language, and visuospatial domains were created.

### 2.4 Statistical analysis

Given the two different acquisition protocols, we first examined the impact of acquisition time on regional SUVR using all [18F]PI-2620 data including those from non-AD participants. Linear mixed models with a random intercept and fixed effects of post-injection time, regional tau status, and post-injection time x regional tau status were conducted for each brain region separately. Post-injection time, centered at 60 min, was the only time-varying term. Second, we combined acquired 45–75 min and interpolated 45–75 min SUVRs and evaluated the pattern of regional tau positivity in Aβ- CU, Aβ+ CU, Aβ+ MCI, and Aβ+ AD Dementia subgroups. We also examined SUVR differences between diagnostic groups and SUVR associations with age and sex. Third, to better understand [18F]PI-2620 signal for early detection of AD, we ran a series of linear regression models with Braak SUVRs predicting CSF pTau-181 after adjusting for amyloid status (Aβ-, Aβ+), age, and sex in a subset of CU participants with available CSF data (Table 2B). Finally, we examined ADRC participants on the aging and AD clinical spectrum who had a neuropsychological assessment within 2 years of their [18F]PI-2620 scan to understand whether Braak SUVRs predicts cognition after controlling for diagnostic group, age, sex, and education (Table 2C); when examining the entire aging and AD clinical spectrum, linear regression was used; when examining CU participants only, robust linear regression using a regression SDMD-estimator via the lmrob function in the robustbase package was used given the small sample size and presence of outliers. Regional analyses performed across aparc+aseg regions were False Discovery Rate (FDR) corrected for multiple comparisons.

Additional methods information regarding participants, amyloid status, regions of interest, tau status, and cognition can be found in Supplementary Materials.

## 3 Results

### 3.1 Timing effects in CU, MCI, AD dementia, and non-AD participants

**Fig. 1.**
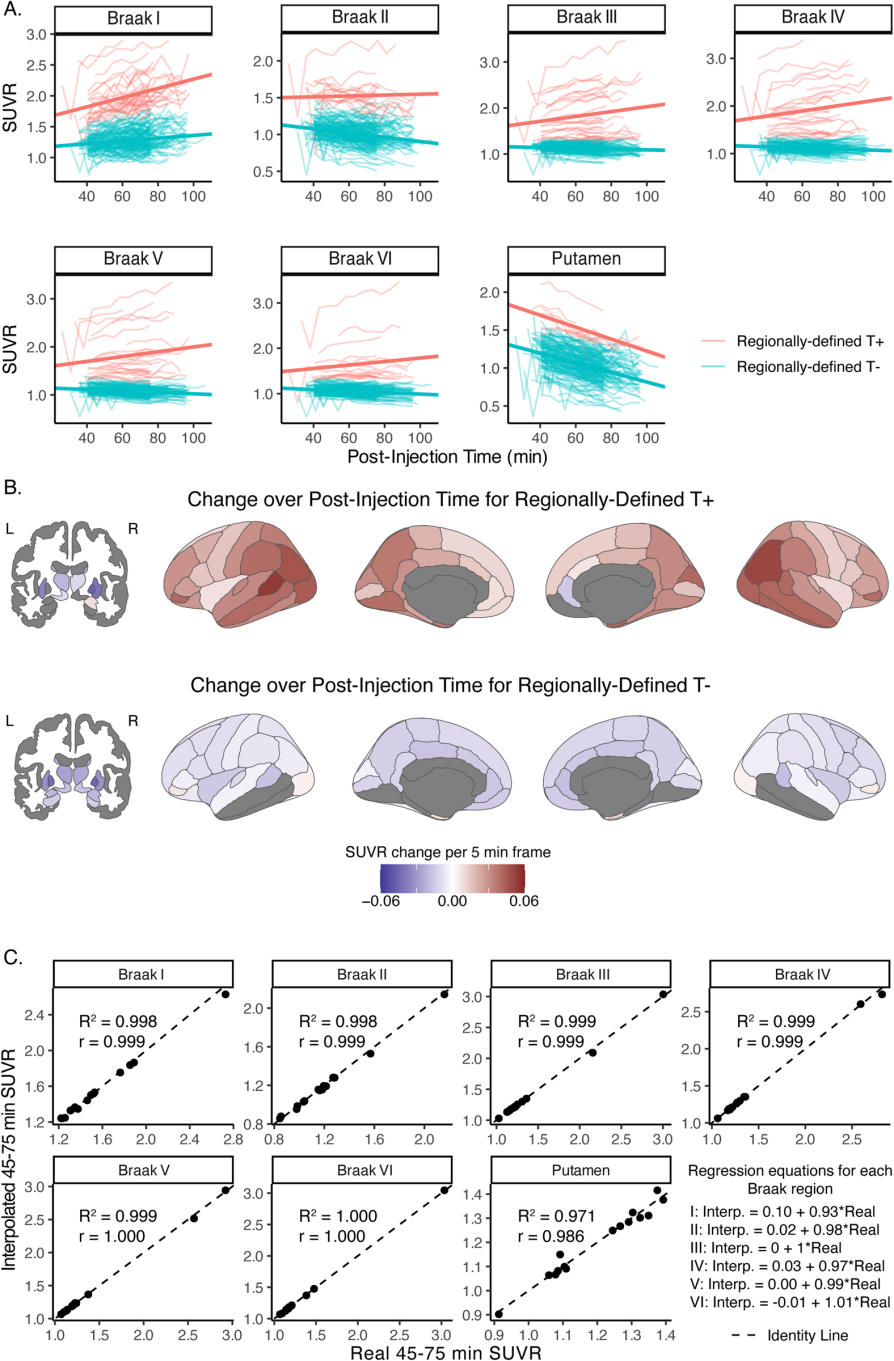
Change in SUVR over acquisition time for regionally defined T- and T+ participants using all data (n = 178) in (A) focused regions of interest and (B) across the whole brain, as well as (C) validation of the interpolation approach adjusting 60–90 min SUVRs to the 45–75 min scale in 15 participants with 45–90 min data. In A and B, linear mixed models with a random intercept and fixed effects of post-injection time, regional tau status, and post-injection time x regional tau status were conducted for each brain region separately. In A, individual data (thin lines) as well as model fits for regionally defined T- and T+ groups (thick lines) are shown. In B, regions with significant (p < 0.05 with FDR correction) change in SUVR over time are shown in blue to red scale; non-significant regions as well as ventricle, brainstem, and corpus callosum regions that were not examined are shown in gray. In C, linear regression and Pearson correlations are shown for SUVRs calculated from 45–75 min data in comparison with SUVRs interpolated to the 45–75 min scale from 60–90 min data (Interpolated 45–75 min SUVR_i_= B_0_+ B_1_* Real 60–90 min SUVR_i_+ ε_i_).

Dynamic frame data showed linear changes in SUVR over time across all six Braak ROIs and in putamen (Fig. 1A). For regionally defined T- participants, SUVRs significantly increased over post-injection time in Braak I, but significantly decreased over post-injection time in Braak II–VI and in putamen (Table 3). In contrast, for regionally defined T+ participants, SUVRs significantly increased over post-injection time in all Braak regions except Braak II, and significantly decreased over post-injection time in putamen.

**Table 3.** Change in SUVR (inferior cerebellum reference region) over acquisition time for regionally defined T- and T+ participants using all data (n = 178).

	T- SUVR at 60 min post-injection	T- change in SUVR per 5 min frame	T+ SUVR at 60 min post-injection	T+ change in SUVR per 5 min frame
Braak I	1.269 (0.016)	0.011 (0.001)	1.970 (0.031)	0.038 (0.002)
Braak II	1.020 (0.011)	-0.014 (0.001)	1.523 (0.031)	0.003 (0.002)
Braak III	1.125 (0.018)	-0.004 (0.001)	1.816 (0.039)	0.026 (0.002)
Braak IV	1.118 (0.017)	-0.006 (0.001)	1.895 (0.040)	0.027 (0.002)
Braak V	1.082 (0.016)	-0.007 (0.001)	1.797 (0.039)	0.025 (0.002)
Braak VI	1.056 (0.013)	-0.008 (0.001)	1.630 (0.039)	0.019 (0.002)
Putamen	1.068 (0.013)	-0.032 (0.001)	1.538 (0.052)	-0.039 (0.003)

Linear mixed models with a random intercept and fixed effects of post-injection time, regional tau status, and post-injection time x regional tau status were conducted for each brain region separately. Note: T+ definitions were based on SUVRs for each region using a cutoff of greater than or equal to 2 standard deviations above the mean of the Aβ- CU group (n = 49). All variables are significantly different than 0 (p < 0.05) except for T+ change in SUVR per 5-min frame in Braak II.

We next examined SUVR change over post-acquisition time in regionally defined T- and T+ groups across aparc+aseg brain regions (Fig. 1B). For regionally defined T- participants, SUVRs generally decreased across cortical and subcortical regions with increasing post-injection time. In contrast, for regionally defined T+ participants, SUVRs significantly increased over time in hippocampus, amygdala, and cortical regions, with the largest increases observed in parietal and occipital regions. Together, these results highlight that acquisition time significantly impacts SUVR quantifications in a manner that is dependent on regional tau status.

### 3.2 [18F]PI-2620 signal across the aging and AD clinical spectrum

Given that our [18F]PI-2620 dataset was acquired using two different acquisition protocols (45–75 min, 60–90 min) and that we demonstrated linear changes in SUVR according to post-injection time, we next adjusted the 60–90 min data to be on the 45–75 min scale using voxel-wise methods described byPontecorvo et al. (2019). In the 15 participants who had data spanning both 60–90 min and 45–75 min post-injection, interpolated SUVRs were highly consistent with SUVRs derived from 45–75 min summed data, demonstrating the validity of this linear interpolation method (Fig. 1C). Thus, all subsequent results used a combination of acquired 45–75 min SUVRs and interpolated 45–75 min SUVRs derived from 60–90 min data (Supplementary Fig. S2).

**Fig. 2.**
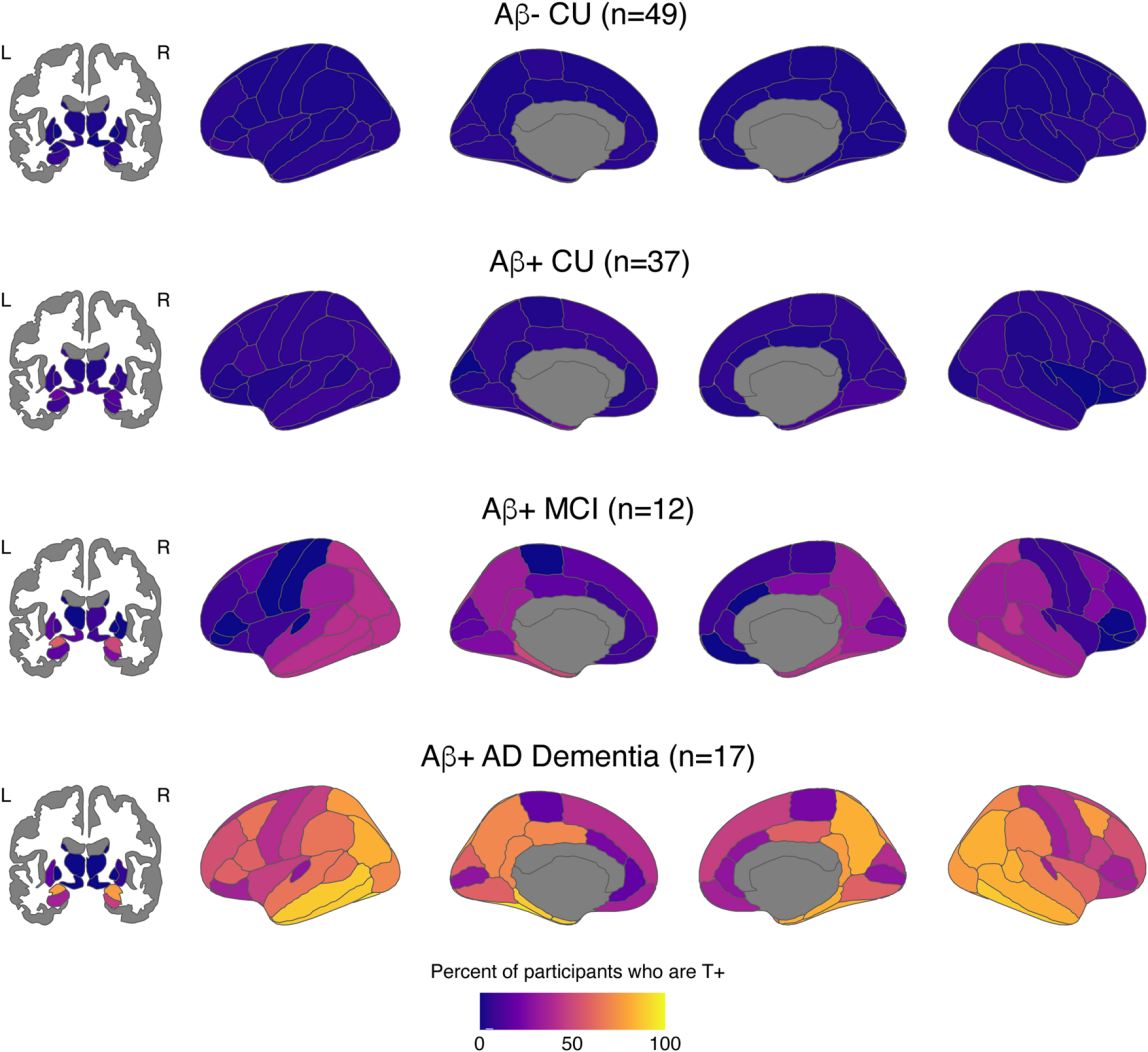
Percent of participants in each diagnostic group who are T+ increases with disease severity. Note: T+ definitions were based on the SUVR for each region using a cutoff of greater than or equal to 2 standard deviations above the mean of the Aβ- CU group (n = 49).

**Fig. 3.**
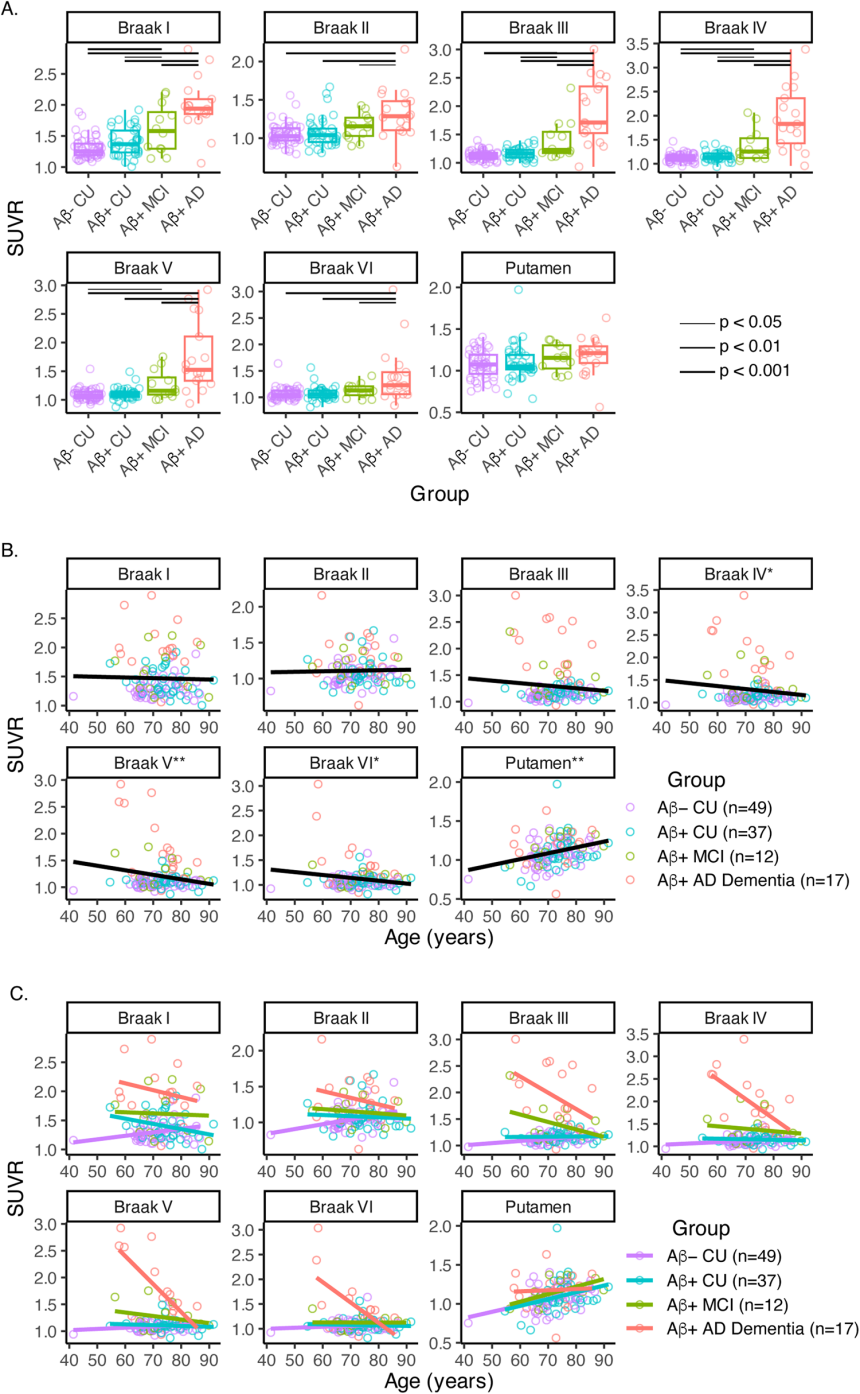
Effects of (A) disease severity, (B) age across diagnostic groups, and (C) age within diagnostic groups on [18F]PI-2620 SUVRs. In A, linear regression models predicting regional tau SUVR were examined (Regional SUVR_i_= B_0_+ B_1_*Diagnostic Group_i_+ B_2_*Age_i_+ B_3_*Sex_i_+ ε_i_); raw SUVR values are shown but statistical analyses accounted for effects of age and sex, and significant differences between diagnostic groups are denoted by horizontal lines. In B, raw values are shown but the same linear regression models were used. In C, raw values are shown but linear regression models were run separately for each diagnostic group.

**Fig. 4.**
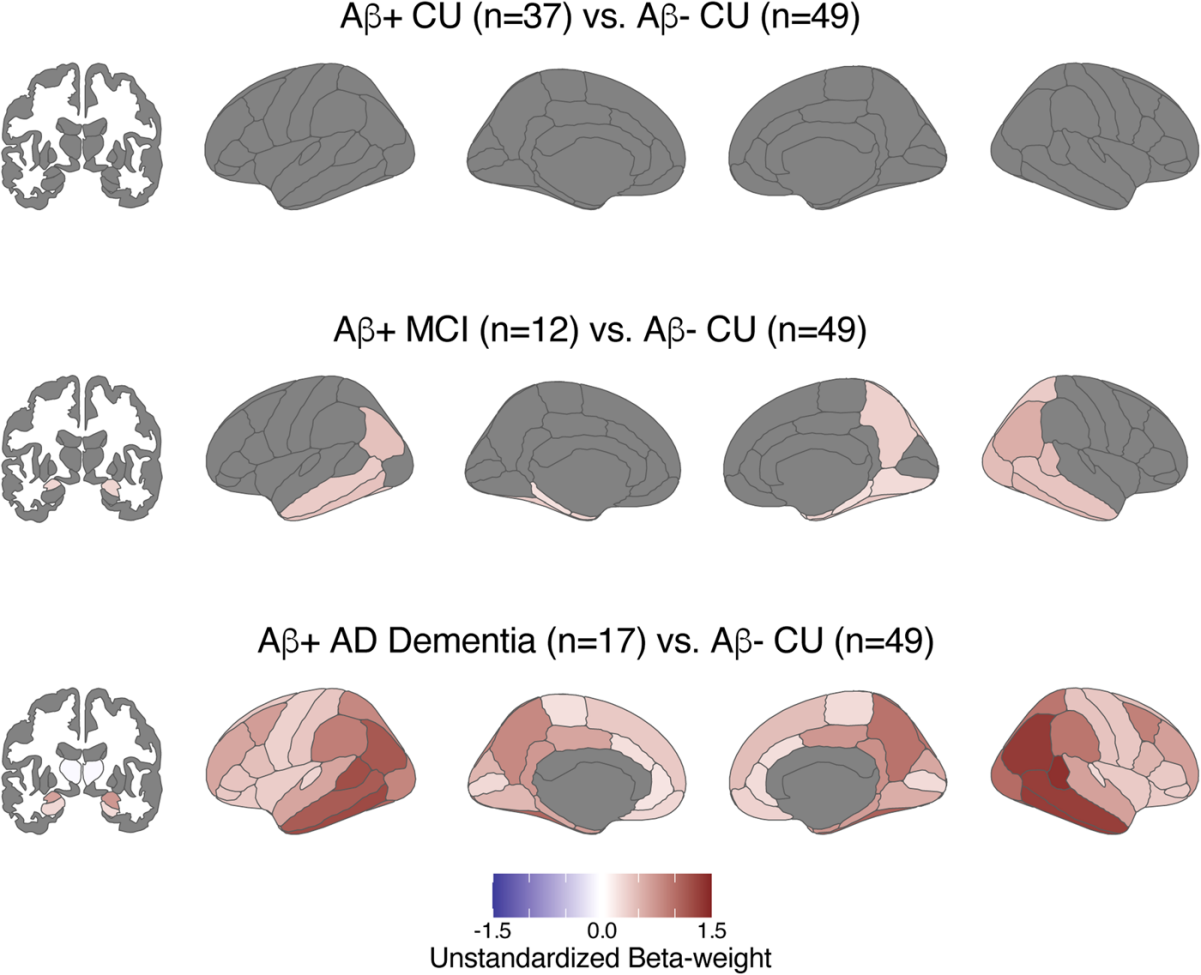
Effects of disease severity on [18F]PI-2620 SUVRs. Linear regression models predicting regional tau SUVR were examined (Regional SUVR_i_= B_0_+ B_1_*Diagnostic Group_i_+ B_2_*Age_i_+ B_3_*Sex_i_+ ε_i_); regions with significant (p < 0.05 with FDR correction) effects are shown in blue to red scale; non-significant regions as well as ventricle, brainstem, and corpus callosum regions that were not examined are shown in gray.

When looking at regional tau positivity across aparc+aseg regions in the aging and AD clinical spectrum, the percent of T+ participants in each region rose with disease severity as expected (Fig. 2). Regional T+ was observed in 0–8% (n = 0–4 out of 49) of Aβ- CU, 0–24% (n = 0–9 out of 37) of Aβ+ CU, 0–58% (n = 0–7 out of 12) of Aβ+ MCI, and 0–94% (n = 0–16 out of 17) of Aβ+ AD Dementia individuals depending on the region. Within the Braak ROIs, SUVRs significantly increased in a stepwise manner with worsening disease severity (Fig. 3A;Supplementary Fig. S3A) and the overall pattern was similar between those with SUVRs calculated from 45–75 min data and those with interpolated SUVRs (Supplementary Fig. S4). There was no difference between disease severity groups in putamen SUVR. Across the whole brain, comparisons between diagnostic groups showed greater SUVRs in Aβ+ MCI compared with Aβ- CU in medial and inferior temporal as well as inferior parietal regions, and greater SUVRs across the whole brain in Aβ+ AD compared with Aβ- CU (Fig. 4).

**Fig. 5.**
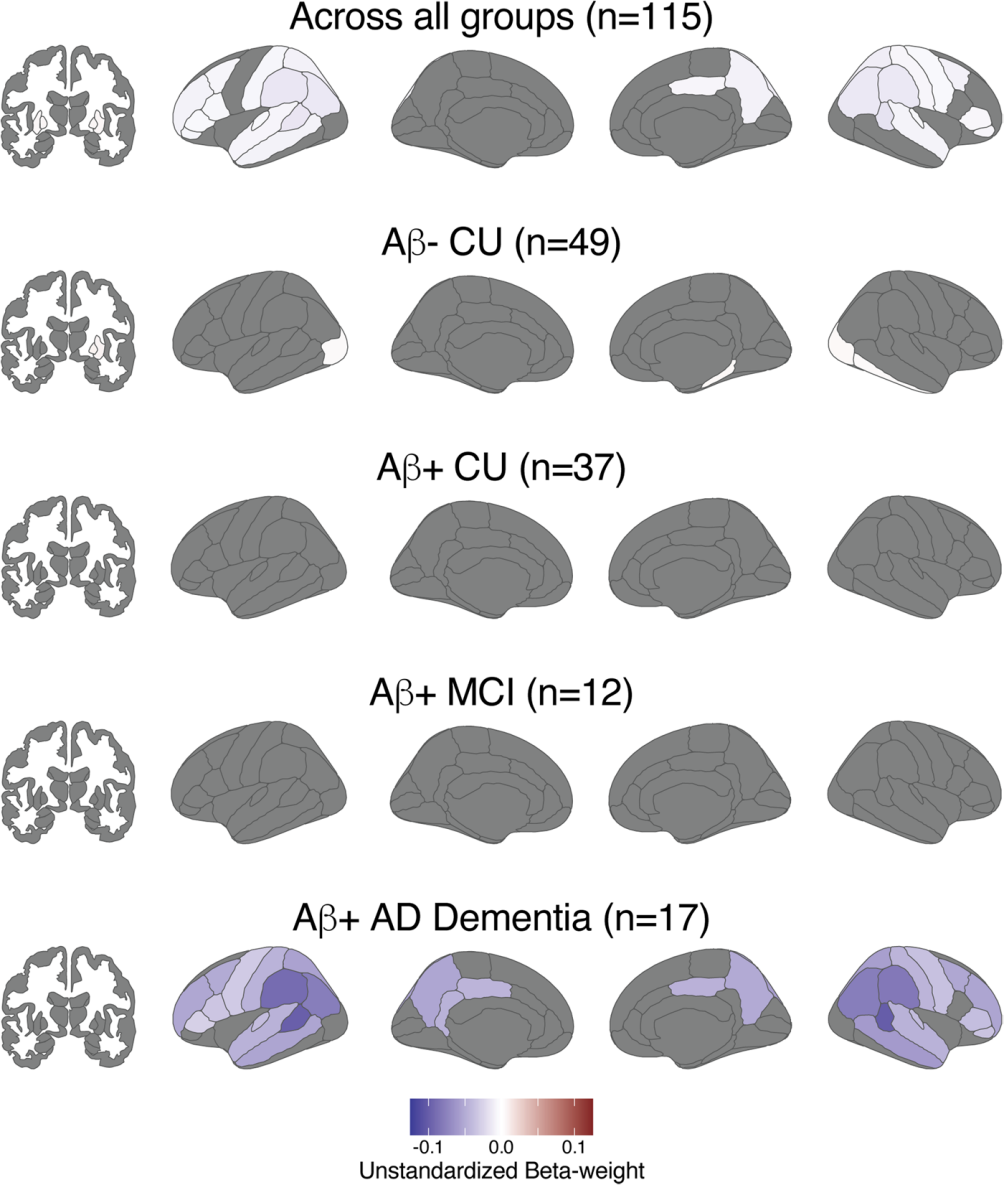
Effects of age on [18F]PI-2620 SUVRs across all groups and within each diagnostic group. Linear regression models predicting regional tau SUVR were examined (across all groups: Regional SUVR_i_= B_0_+ B_1_*Diagnostic Group_i_+ B_2_*Age_i_+ B_3_*Sex_i_+ ε_i_; within each group: Regional SUVR_i_= B_0_+ B_1_*Age_i_+ B_2_*Sex_i_+ ε_i_); regions with significant (p < 0.05 with FDR correction) effects are shown in blue to red scale; non-significant regions as well as ventricle, brainstem, and corpus callosum regions that were not examined are shown in gray.

When examining all participants across the aging and AD clinical spectrum, older age was significantly associated with lower SUVRs in Braak IV–VI regions and higher SUVRs in putamen (Fig. 3B;Supplementary Fig. S3B;Supplementary Table S1). Within each diagnostic group, older age was associated with higher SUVR in Braak I–III within the Aβ- CU group, and lower SUVR in Braak IV–VI within the Aβ+ AD Dementia group (Fig. 3C;Table 4;Supplementary Fig. S3C;Supplementary Table S2). Older age was also associated with increasing putamen SUVR, though this was only significant in the Aβ- CU group. Across all brain regions and all aging and AD clinical spectrum participants, older age similarly showed significant negative associations with SUVR in cortical regions (Fig. 5). Within diagnostic groups, age effects were only widely prominent in the Aβ+ AD Dementia group. There were no significant sex effects in focused regions of interest or across all brain regions when examining all participants across the aging and AD clinical spectrum or when examining each diagnostic group individually.

**Table 4.** Age and sex effects on regional [18F]PI-2620 SUVRs within each diagnostic group.

	Age	Sex (M vs. F)
Within Aβ- CU (n = 49)		
Braak I	**0.006 (0.003), p** **=** **0.048**	0.088 (0.048), p = 0.075
Braak II	**0.007 (0.002), p** **=** **0.008**	0.041 (0.039), p = 0.304
Braak III	**0.004 (0.001), p** **=** **0.006**	0.023 (0.023), p = 0.309
Braak IV	0.003 (0.002), p = 0.065	0.048 (0.025), p = 0.059
Braak V	0.002 (0.002), p = 0.210	0.038 (0.029), p = 0.200
Braak VI	0.002 (0.002), p = 0.340	0.051 (0.034), p = 0.137
Putamen	**0.008 (0.003), p** **=** **0.004**	-0.029 (0.044), p = 0.514
Within Aβ+ CU (n = 37)		
Braak I	-0.009 (0.005), p = 0.082	0.006 (0.076), p = 0.940
Braak II	-0.002 (0.005), p = 0.716	0.005 (0.069), p = 0.941
Braak III	-0.000 (0.002), p = 0.954	0.044 (0.034), p = 0.201
Braak IV	-0.002 (0.002), p = 0.403	0.053 (0.033), p = 0.115
Braak V	-0.002 (0.003), p = 0.400	0.053 (0.037), p = 0.164
Braak VI	-0.002 (0.003), p = 0.497	0.079 (0.043), p = 0.079
Putamen	0.008 (0.005), p = 0.118	0.065 (0.073), p = 0.382
Within Aβ+ MCI (n = 12)		
Braak I	-0.002 (0.014), p = 0.909	-0.034 (0.268), p = 0.903
Braak II	-0.002 (0.006), p = 0.695	-0.062 (0.115), p = 0.603
Braak III	-0.013 (0.011), p = 0.292	-0.134 (0.225), p = 0.565
Braak IV	-0.007 (0.011), p = 0.579	0.118 (0.224), p = 0.610
Braak V	-0.007 (0.009), p = 0.414	0.072 (0.168), p = 0.680
Braak VI	0.000 (0.005), p = 0.960	-0.036 (0.093), p = 0.706
Putamen	0.010 (0.005), p = 0.051	-0.071 (0.091), p = 0.454
Within Aβ+ AD Dementia (n = 17)		
Braak I	-0.011 (0.014), p = 0.411	-0.055 (0.225), p = 0.809
Braak II	-0.009 (0.010), p = 0.406	-0.104 (0.168), p = 0.547
Braak III	-0.028 (0.015), p = 0.080	-0.415 (0.249), p = 0.118
Braak IV	**-0.043 (0.017), p** **=** **0.023**	-0.125 (0.280), p = 0.663
Braak V	**-0.052 (0.014), p** **=** **0.002**	-0.050 (0.227), p = 0.827
Braak VI	**-0.040 (0.013), p** **=** **0.008**	-0.279 (0.211), p = 0.207
Putamen	0.002 (0.007), p = 0.787	-0.091 (0.122), p = 0.470

Each row represents a separate model (Regional SUVR_i_= B_0_+ B_1_*Age_i_+ B_2_*Sex_i_+ ε_i_) and unstandardized betas (standard error) and p values are provided. Significant effects are shown in bold text.

### 3.3 [18F]PI-2620 associations with CSF pTau-181 in Aβ- CU and Aβ+ CU

**Fig. 6.**
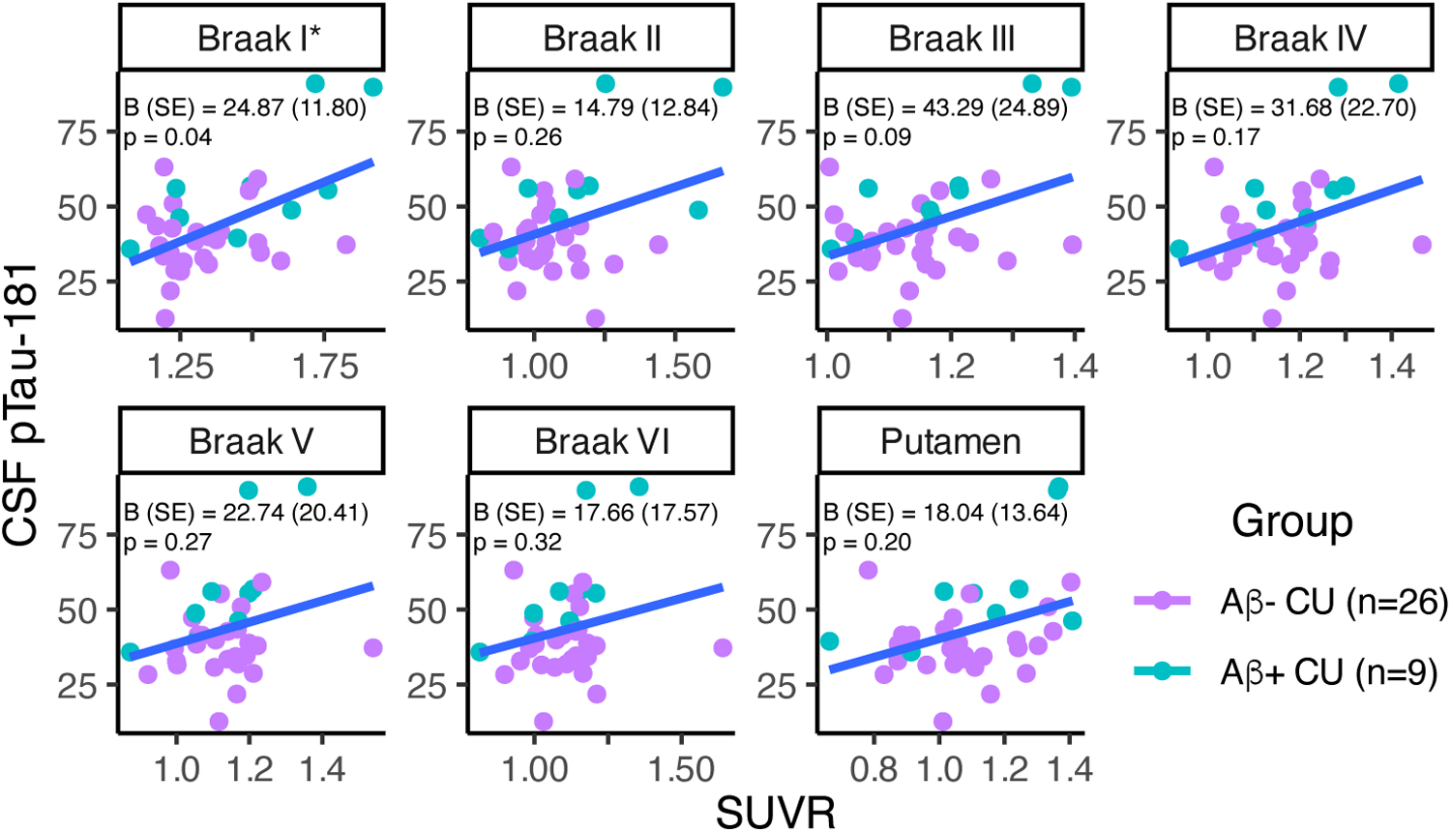
Associations between [18F]PI-2620 SUVR and CSF pTau-181 in 35 cognitively unimpaired (CU) participants. SUVR and CSF pTau-181 values are shown but statistical analyses accounted for effects of amyloid status, age, and sex (linear regression model: CSF pTau-181_i_= B_0_+ B_1_*Amyloid Status_i_+ B_2_*Age_i_+ B_3_*Sex_i_+ ε_i_). Unstandardized betas (standard error) and p values are provided. Note: *p < 0.05.

In a subset of 35 CU participants with CSF pTau-181 data (Table 2B), greater Braak I SUVR was significantly associated with greater CSF p-Tau181 (Fig. 6;Supplementary Fig. S5). Similar patterns were observed in Braak II–VI and putamen, though results were non-significant.

### 3.4 [18F]PI-2620 associations with cognition across the aging and AD clinical spectrum

**Fig. 7.**
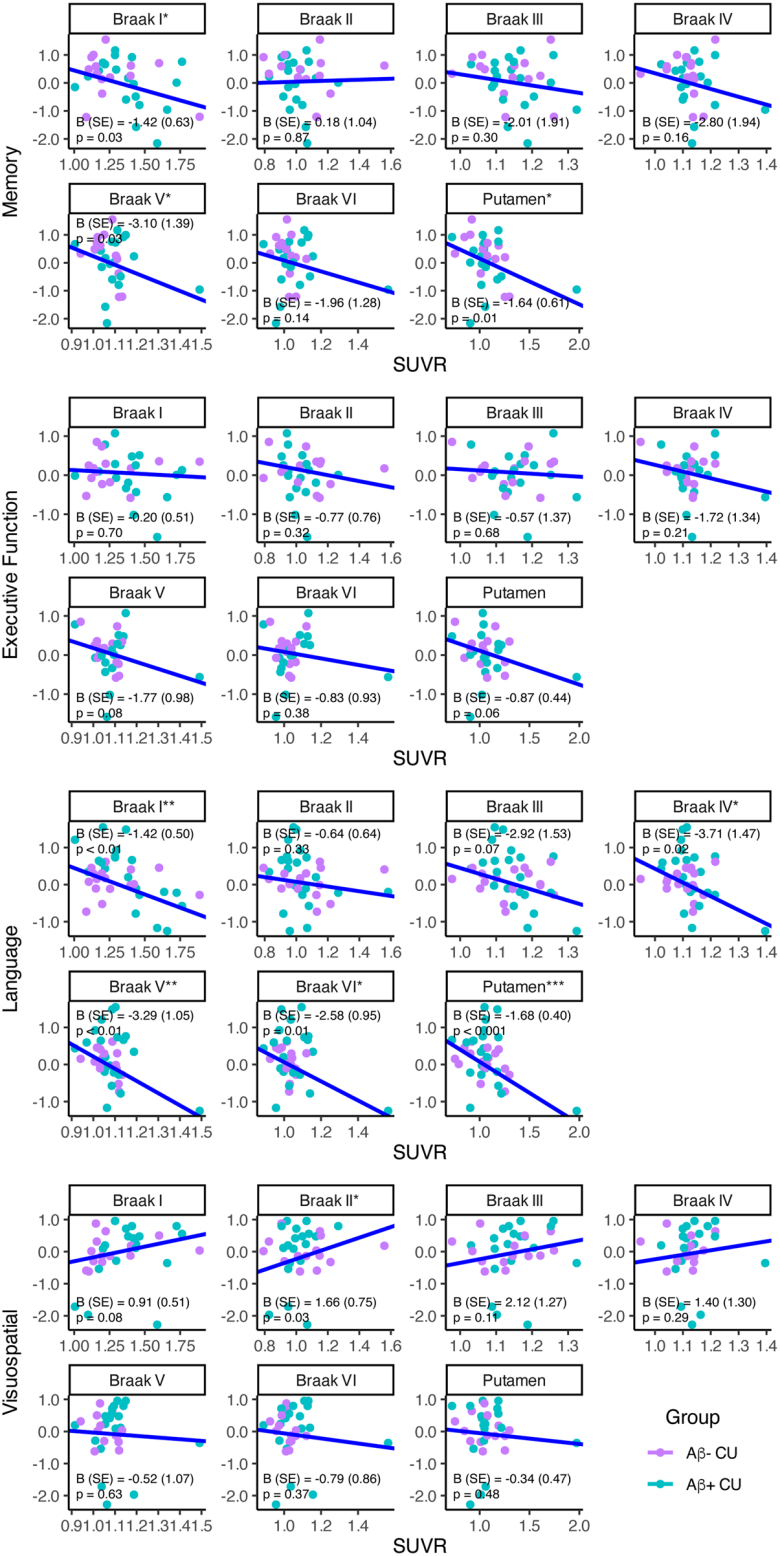
[18F]PI-2620 SUVR associations with cognitive composites in 37 cognitively unimpaired (CU) participants. Raw SUVR, raw cognitive composite scores, and robust linear regression fits when controlling for amyloid status, age, sex, and education are shown (Cognition_i_= B_0_+ B_1_*Amyloid Status_i_+ B_2_*Age_i_+ B_3_*Sex_i_+ B_4_*Education_i_+ ε_i_). Robust regression unstandardized betas (standard error) and p values are provided. Note: *p < 0.05, **p < 0.01, ***p < 0.001.

In a subset of 57 participants with available neuropsychological data (Table 2C), there were strong significant effects of diagnostic group, but limited effects of regional tau on cognition (Supplementary Tables S3 and S4). When examining CU participants only with robust regression, greater SUVR was significantly and most consistently associated with reduced memory and language (Fig. 7).

## 4 Discussion

Through examination of dynamic [18F]PI-2620 data collected with two common acquisition protocols, we demonstrated the impact of post-injection time on SUVR quantification. For participants with substantial amounts of tau (T+), SUVRs increased linearly with post-injection time, and in Braak composite regions, SUVRs increased up to 0.04 with each additional 5 min past injection time. Additionally, we demonstrated the validity of adjusting SUVRs to the recommended 45–75 min timeframe using a participant-level voxel-wise linear regression approach. Our resulting dataset that combined acquisition protocols showed the expected increases in [18F]PI-2620 signal with worsening AD disease severity. Finally, we highlighted the potential of [18F]PI-2620 for early detection of AD as greater [18F]PI-2620 signal was associated with greater CSF pTau-181 and worse memory and language in CU participants.

As tau PET ligands are first integrated into the research setting, it is common for acquisition windows to differ across initial datasets before recommended research windows are decided. [18F]PI-2620 has been evaluated in the context of AD across a broad imaging window of 30–90 min post-injection (Bullich et al., 2020;Mueller et al., 2020) and some work has suggested that analysis of dynamic data spanning 0–60 min is ideal for capturing subcortical signal associated with 4R tauopathies (Brendel et al., 2020) with later time windows showing better discrimination from healthy controls (Blazhenets et al., 2023). Thus, multiple scanning protocols for [18F]PI-2620 currently exist depending on the intended clinical use. Our results, which converge with reports that [18F]PI-2620 SUVRs can continue to increase until 180 min post-injection without stabilization for some AD participants with presumably the highest levels of tau (Bullich et al., 2020;Mueller et al., 2020), highlight the importance of adjusting for protocol differences to combine datasets. This will be especially important to consider when assessing the effects of AD treatments on tau burden (Edwards et al., 2023), as protocol differences in acquisition time before and after treatment can obscure detection of true changes in tau burden or lead to spurious findings.

We show that a voxel-wise linear regression approach in which each voxel’s intercept and slope over time are used to adjust SUVRs to the currently recommended 45–75 min timescale yields adjusted SUVRs that are highly consistent with SUVRs based on data collected 45–75 min post-injection. Our results also caution against directly comparing SUVRs from studies with different acquisition protocols as studies with later acquisition times will likely report higher SUVRs that reflect the protocol rather than higher levels of tau accumulation. The effects of varying acquisition protocols will be especially important to consider as [18F]PI-2620 moves from research to clinical settings, where protocol variance is likely to be much greater across participants and timepoints.

Previous studies with Aβ- or unknown Aβ status in CU and AD participants have shown negative associations between [18F]PI-2620 SUVRs and Mini-Mental State Examination (Bullich et al., 2022;Bun et al., 2022;Mueller et al., 2020;Rullmann et al., 2022), Alzheimer’s Disease Assessment Scale—Cognitive Subscale (ADAS-Cog) (Bun et al., 2022;Mueller et al., 2020), and one-card learning (Bullich et al., 2022) scores. In our study, associations between SUVR and cognition were largely non-significant across all aging and AD clinical spectrum participants when diagnostic group was included in the model. This finding highlights that cognition is best predicted by diagnostic status (which occurs because cognitive performance partially defines diagnostic status) and that tau does not provide much additional information beyond diagnostic group when predicting cognition across the aging and AD clinical spectrum. However, when examining CU participants only, SUVRs across Braak ROIs were most consistently related to memory and language performance, demonstrating that [18F]PI-2620 is able to capture tau aggregates and can provide meaningful information early in disease.

We additionally identified significant negative age associations, particularly in parietal and lateral temporal regions, that were driven by the Aβ+ AD Dementia group. This inverse age relationship replicates previous studies examining tau burden with FTP (La Joie et al., 2021;Ossenkoppele et al., 2021) and converges with findings from longitudinal tau PET studies that have shown increases in tau burden over time in both early onset and sporadic AD (Jack et al., 2018;Sanchez et al., 2021;Sintini et al., 2019). The negative age association likely reflects a combination of more pure and aggressive AD pathology in younger AD Dementia participants (Gerritsen et al., 2016) and less age-related co-pathology in younger patients, which would then require greater levels of tau burden to reach a similar level of impairment compared with their older counterparts.

First-generation tau PET tracers, including FTP, were known to have significant off-target binding in choroid plexus, which obscured the ability to examine tau in adjacent hippocampus (Lowe et al., 2016), as well as in basal ganglia regions including putamen (Choi et al., 2018;Lockhart et al., 2017;Lowe et al., 2016). Off-target binding with [18F]PI-2620 has not yet been fully characterized and it is currently unclear whether hippocampal (Braak II) [18F]PI-2620 signal can be interpreted. Our study shows that hippocampal [18F]PI-2620 SUVRs increase with worsening disease progression and do not show significant age associations across the aging and AD clinical spectrum. However, greater hippocampal SUVR was significantly associated with older age within Aβ- CU only; was not significantly associated with CSF pTau-181 in CU participants; and was not significantly associated with cognition in CU participants (except in the opposite than expected direction such that higher hippocampal tau was associated with better visuospatial performance). Thus, it remains unclear whether hippocampal signal extracted with [18F]PI-2620 provides important biological information beyond what can be achieved when examining other Braak regions. With respect to basal ganglia, previous studies have reported a lack of off-target binding in these regions (Mormino et al., 2021). However, we found age-related increases in putamen when all participants across the aging and AD clinical spectrum were examined together, similar to what has been reported with FTP (Choi et al., 2018). The lack of significant age-related increases in putamen within Aβ+ CU, Aβ+ MCI, and Aβ+ AD Dementia groups likely reflects the small sample size within each diagnostic group as the regression beta weights were largely consistent though non-significant. We also showed that putamen SUVRs did not significantly increase with disease severity, and was not significantly associated with CSF pTau-181 in CU. Direct comparisons of off-target binding are needed to understand whether off-target basal ganglia binding is diminished with [18F]PI-2620 in comparison with FTP.

There are several limitations to consider. First, although our overall sample size consisted of 178 participants including 115 participants on the healthy aging and AD clinical spectrum, our MCI and AD Dementia groups and our CSF pTau-181 and cognitive subsets were relatively small. We also did not adjust for multiple comparisons except when examining all aparc+aseg regions and replication with larger samples are needed. Second, our cohort consisted of primarily non-Hispanic White participants and the generalizability of these findings to participants of other ethnic and racial backgrounds with different comorbidities is unknown. Third, [18F]PI-2620 SUVRs were calculated with an inferior cerebellum reference region and other reference regions may be better for detecting group differences and associations with cognition. Relatedly, we interpolated data from 60–90 min post-injection to a 45–75 min scale given current recommendations for [18F]PI-2620 (Bullich et al., 2020;Mueller et al., 2020). Given that SUVR values are lower during this earlier acquisition window, effect sizes between high and low tau participants may be underestimated compared with data collected 60–90 min post-injection. However, we nonetheless found the expected pattern of results as a function of disease stage and cognition, providing support for the use of this earlier acquisition scale. Finally, partial volume correction was not applied.

## 5 Conclusion

[18F]PI-2620 data collected across different acquisition protocols will need to be corrected for timing differences. After correcting for post-injection time, [18F]PI-2620 shows the expected increases across the aging and AD clinical spectrum and is associated with cognitive deficits. This work provides further evidence that [18F]PI-2620 accurately captures tau aggregations in AD.

## Data and Code Availability

Data collected through the ADRC are shared with and available through the National Alzheimer’s Coordinating Center (NACC). Data collected through the Stanford and Aging Memory Project (SAMS) and the Stanford Center for Memory Disorders will be made available following a formal data sharing agreement with the senior author, Elizabeth C. Mormino. Code will also be made available upon request.

## Author Contributions

Christina B. Young: Conceptualization, methodology, formal analysis, writing, visualization. Hillary Vossler and America Romero. Investigation, data curation, project administration. Viktorija Smith: Data curation. Jennifer Park: Investigation, data curation. Alexandra N. Trelle, Joseph R. Winer, Michael M. Zeineh, Mehdi Khalighi, Aimara P. Morales, David Anders, Greg Zaharchuk, Victor W. Henderson, Katrin I. Andreasson, Anthony D. Wagner, Kathleen L. Poston, and Guido A. Davidzon: Writing—review & editing. Edward N. Wilson and Sharon J. Sha: Investigation, writing—review & editing. Maya V. Yutsis: Investigation. Elizabeth C. Mormino: Conceptualization, writing—review & editing, supervision.

## Funding

This study was supported by grants K99AG071837, K99AG075184, R01AG048076, R01AG074339, R21AG058859, P30AG066515, R01NS115114 from the National Institute of Health; AARFD-21-849349 from the Alzheimer’s Association; Wu Tsai Neuroscience Institute; Precision Health and Integrated Diagnostics Center at Stanford; and the Good Planet Foundation.

## Declaration of Competing Interest

Dr. Poston is a paid consultant for CuraSen Therapeutics and Biohaven Pharmaceuticals. Dr. Mormino is a paid consultant for Roche, Genentech, Eli Lilly, and Neurotrack. All other authors have no disclosures.

## Supplementary Materials

Supplementary material for this article is available with the online version here:https://doi.org/10.1162/imag_a_00329.

## Supplementary Material

Supplementary Material
