# Supplementary material for: [18F]PI-2620 Tau PET signal across the aging and Alzheimer’s disease clinical spectrum
Source: Imaging Neurosci (Camb). 2024 Oct 24;2:imag-2-00329. doi: 10.1162/imag_a_00329 (PMC12290608; doi:10.1162/imag_a_00329)
Supplement: Supplementary Material [file imag_a_00329-supp.pdf]

10. Supplementary Materials

10.1. Supplementary Methods

10.1.1. Participants

Stanford ADRC participants underwent standardized neurological, cognitive, and laboratory assessments and were reviewed by a panel including neurologists and neuropsychologists who determined the cognitive status and presumptive etiology for participants with cognitive impairment (Shahid et al., 2023). The neuropsychological battery for Stanford ADRC participants consisted of the National Alzheimer's Coordinating Center Uniform Data Set and supplementary tests [i.e., Judgment of Line Orientation, Hopkins Verbal Learning Test – Revised (HVLT-R), Selective Reminding Cued Recall Test, Letter-Number Sequencing, Clock Drawing Test, Victoria Stroop Test, Cookie Theft, Wechsler Test of Adult Reading (WTAR)]. SAMS is an NIH-funded imaging-biofluid observational cohort study of normal aging that began enrolling in 2014 (Trelle et al., 2020, 2021). All SAMS participants were 60-90 years of age at baseline enrollment and classified as CU at a clinical consensus meeting by a panel of neurologists and neuropsychologists. The neuropsychological battery for SAMS participants consisted of the Wechsler Memory Scale 3rd Edition (WMS-III) Logical Memory, HVLT-R, Brief Visuospatial Memory Test – Revised (BVMT-R), Animal Fluency, Controlled Oral Word Association Test (COWAT), Trail Making Test, Digit Symbol Substitution, WMS-III Digit Span, Boston Naming Test – 30 item (BNT-30), Wechsler Abbreviated Scale of Intelligence (WASI) Similarities, WASI Block Design, WASI Matrix Reasoning, Repeatable Battery for the Assessment of Neuropsychological Status (RBANS) Update Figure Copy, WTAR, Montreal Cognitive Assessment, and Geriatric Depression Scale (GDS). For Stanford Center for Memory Disorders participants, diagnosis was determined by the treating neurologist. Neuroimaging and CSF biomarker data was used to inform clinical diagnoses when available. Diagnosis at the time closest to the tau PET scan was used and all included participants had a diagnosis within 2 years of the tau PET scan. All participants

provided written informed consent or assent, and protocols were approved by the Stanford institutional review board.

The 54 non-AD participants included 17 Parkinson's disease (PD) without cognitive impairment, 2 MCI due to corticobasal degeneration, 1 MCI due to cognitive disorder not otherwise specified, 1 MCI due to cerebrovascular disease (CVD), 10 MCI due to lewy body disease (LBD), 8 MCI due to PD, 1 MCI due to post-traumatic stress disorder, 1 MCI due to traumatic brain injury, 2 dementia due to CVD, 1 dementia due to frontotemporal lobar degeneration, 5 dementia due to LBD, 1 dementia due to traumatic brain injury, 1 corticobasal syndrome, 1 progressive supranuclear palsy, and 2 semantic variant primary progressive aphasia participants.

<u>10.1.2. Amyloid Status</u>

165 participants had amyloid PET, amyloid CSF and/or plasma amyloid data available. If multiple amyloid datapoints were available, amyloid data closest to the [18F]PI-2620 scan were considered. Participants with an amyloid status had amyloid data collected within 3 years of the [18F]PI-2620 scan date, or positive amyloid statuses more than 3 years before the [18F]PI-2620 scan (i.e., negative amyloid statuses that were more than 3 years before the [18F]PI-2620 scan were excluded since the participant may have become amyloid positive in the interim time).

10.1.2.1. Amyloid PET: Amyloid status was determined by amyloid PET for 99 participants. Amyloid PET scanning was completed at the Richard M. Lucas Center for Imaging at Stanford University, using a simultaneous time-of-flight (TOF)–enabled PET/MRI scanner (SIGNA 3T, GE HealthCare). Emission data were collected between 90-110 min following an 8.1mCi injection of ^{18}F-florbetaben (FBB). PET data were reconstructed into 5-min frames using standard methods with zero-TE (ZTE) or Dixon MR imaging for MR attenuation correction. 5-min frame data were

realigned and summed. FreeSurfer v7 ROIs from the Desikan aparc+aseg atlas were defined on each participant's MRI [high-resolution T1-weighted spoiled gradient recalled echo (SPGR) scan, TR = 7.664 ms, TE = 3.09 ms, TI = 400 ms, flip angle = 11, 1.2 × 1.1 × 1.1 mm]. The native space FreeSurfer ROIs were used to extract intensity values from the co-registered summed amyloid PET data. Preprocessing followed ADNI procedures; the standardized uptake value ratio (SUVR) was calculated for a global cortical ROI using a whole cerebellum reference region, centiloids were calculated using the equation from Royse et al. (2021) ($CL_{whole\ cerebellum} = 157.15 \times SUVR_{FBB} - 151.87$), and a cutoff of 18 centiloids was used to determine amyloid positivity (Royse et al., 2021).

10.1.2.2. CSF Aβ42/Aβ40*:* Amyloid status was determined by CSF for 39 participants. CSF was collected via lumbar puncture in the morning following overnight fasting. CSF was centrifuged at 1500 x g for 15 min at 20°C, aliquoted in polypropylene tubes, and stored at −80°C. A single aliquot was used to measure AD biomarkers including Aβ42 and Aβ40 on the fully automated Lumipulse G system (Fujirebio US, Inc., Malvern, PA) as previously described (Trelle et al., 2021; Wilson et al., 2022). Using a larger dataset of 153 SAMS participants (Trelle et al., 2021), a 2-cluster Gaussian mixture modeling approach was used to define a Aβ42/Aβ40 cutoff of 0.075 based off the 0.5 probability of belonging to the Aβ+ distribution (Trelle et al., 2021).

10.1.2.3. Plasma Aβ42/Aβ40: Amyloid status was determined by plasma Aβ42/Aβ40 for 18 participants. EDTA plasma was collected by venipuncture, centrifuged for 10 minutes at 2000 x g at 22°C, aliquoted in polypropylene tubes, and stored at -80°C until measurement. A single aliquot was used to measure Aβ42 and Aβ40 on the fully automated Lumipulse G system (Fujirebio US, Inc., Malvern, PA) as previously described (Wilson et al., 2022). Using a larger dataset of 196 ADRC and SAMS participants with plasma Aβ42/Aβ40 and CSF Aβ42/Aβ40, the Youden method

was used to determine the optimal plasma Aβ42/Aβ40 cutoff of 0.0943 to maximize sensitivity and specificity in discriminating CSF Aβ+ from Aβ- individuals (Trelle et al., Under Review).

10.1.2.4. Clinical Amyloid Data: A small subset of 9 participants had pre-existing amyloid PET (n = 6) or CSF (n = 3) data collected as part of a clinical trial or clinical care. For these participants, only dichotomous amyloid status (Aβ-, Aβ+) was available.

10.1.3. [18F]PI-2620 Data

PET images were reconstructed using TOF ordered subset expectation maximization (TOF-OSEM) with 3 iterations, 28 subsets, and 2.78 × 1.17 × 1.17 mm voxel size. Corrections were applied for detector deadtime, scatter, randoms, detector normalization, and radioisotope decay. Attenuation correction was performed with ZTE MR imaging for MR attenuation correction [16]. PET data were reconstructed into 5-min frames and frames were realigned and summed. FreeSurfer v7 ROIs from the Desikan aparc+aseg atlas were defined on each participant's MRI (same imaging parameters as the T1-weighted SPGR scan acquired during the amyloid PET scan). The native space FreeSurfer ROIs were used to extract intensity values from the co-registered summed PET data. SUVRs were calculated using an inferior cerebellum reference region defined by the spatially unbiased atlas template (SUIT) toolbox in MATLAB (Diedrichsen et al., 2011).

10.1.3.1. Regions of Interest and tau status: We focused on 6 a priori bilateral volume-weighted regions of interest (ROIs) that mimic Braak stages [Braak I: entorhinal; Braak II: hippocampus; Braak III: parahippocampal, fusiform, lingual, amygdala; Braak IV: middle temporal, caudal anterior cingulate, rostral anterior cingulate, posterior cingulate, isthmus cingulate, insula, inferior temporal, temporal pole; Braak V: bank STS, caudal middle frontal, frontal pole, inferior parietal, lateral occipital, lateral orbitofrontal, pars opercularis, pars orbitalis, pars triangularis, precuneus,

rostral middle frontal, superior frontal, superior parietal, superior temporal, supramarginal, transverse temporal; Braak VI: pericalcarine, postcentral, cuneus, precentral, paracentral] (Biel et al., 2021) as well as the putamen, a region known to have off-target binding with FTP (Choi et al., 2018). We additionally examined [18F]PI-2620 signal across the brain using all aparc+aseg regions except ventricle, brainstem, corpus callosum, and cerebellum regions. Tau positive (T+) status was defined for each brain region as 2 SDs greater than the mean of Aβ- CUs (n=49) using acquired 45-75 min and interpolated 45-75 min data.

10.1.4. Cognition

ADRC participants completed the National Alzheimer's Coordinating Center Uniform Data Set - 3 neuropsychological battery in addition to supplemental cognitive tests. For each neuropsychological test, z-scores were calculated using means and standard deviations of baseline visit from 236 CU participants (i.e., not only participants with PET data) in the ADRC. The z-scores were then used to create domain-specific composite scores by averaging all relevant z-scores for the domain. The memory composite consisted of Craft Story delayed recall, Benson Figure delayed recall, HVLT-R delayed recall, and Free and Cued Selective Reminding Test delayed free recall (requiring at least 2 out or 4 scores). The executive functioning composite consisted of the Trail Making Test (Part A, Part B), Number Span Forward, Number Span Backward, Victoria Stroop (Color Time, Word Time, Color-Word Time), Letter Number Sequencing, Digit Symbol Coding, and Clock Drawing by command (requiring at least 5 out of 10 scores). The language composite consisted of the Multilingual Naming Test (MINT), Phonemic Fluency (F, L), Semantic Fluency (animals, vegetables), and Cookie Theft (requiring at least 2 out of 4 scores). The visuospatial score consisted of Benson Figure copy, Judgment of Line Orientations, and Clock copy (requiring at least 2 out of 3 scores).

10.2. Supplementary Results

10.2.1. Participants

There was a significant difference in recruitment source for each diagnostic group [Fisher's exact test, $p < 0.001$]. More specifically, CU participants were recruited from the Stanford ADRC (n=24; 27%), the SAMS (n=42; 46%), or were co-enrolled in the ADRC and SAMS (n=25; 27%); MCI participants were recruited from the ADRC (n=12; 86%) and through the Stanford Center for Memory Disorders (n=2; 14%); AD Dementia participants were recruited from the Stanford ADRC (n=13; 68%) and the Stanford Center for Memory Disorders (n=6; 32%); and Non-AD participants were recruited from the Stanford ADRC (n=50; 93%) and the Stanford Center for Memory Disorders (n=4; 7%). CU, MCI, AD Dementia, and Non-AD groups did not significantly differ in age [$F(3,174) = 0.587$, $p = 0.625$]. However, the diagnostic groups did significantly differ in sex [$\chi^2(3) = 13.833$, $p = 0.003$] such that the CU and AD Dementia groups consisted of 60-63% females whereas the MCI and Non-AD groups consisted of 64-69% males (**Table 2**).

Supplementary References

Biel, D., Brendel, M., Rubinski, A., Buerger, K., Janowitz, D., Dichgans, M., Franzmeier, N., & for the Alzheimer's Disease Neuroimaging Initiative (ADNI). (2021). Tau-PET and in vivo Braak-staging as prognostic markers of future cognitive decline in cognitively normal to demented individuals. *Alzheimer's Research & Therapy*, *13*(1), 137. https://doi.org/10.1186/s13195-021-00880-x

Choi, J. Y., Cho, H., Ahn, S. J., Lee, J. H., Ryu, Y. H., Lee, M. S., & Lyoo, C. H. (2018). Off-Target 18F-AV-1451 Binding in the Basal Ganglia Correlates with Age-Related Iron Accumulation. *Journal of Nuclear Medicine*, *59*(1), 117–120. https://doi.org/10.2967/jnumed.117.195248

Diedrichsen, J., Maderwald, S., Küper, M., Thürling, M., Rabe, K., Gizewski, E. R., Ladd, M. E., & Timmann, D. (2011). Imaging the deep cerebellar nuclei: A probabilistic atlas and normalization procedure. *NeuroImage*, *54*(3), 1786–1794. https://doi.org/10.1016/j.neuroimage.2010.10.035

Royse, S. K., Minhas, D. S., Lopresti, B. J., Murphy, A., Ward, T., Koeppe, R. A., Bullich, S., DeSanti, S., Jagust, W. J., Landau, S. M., & for the Alzheimer's Disease Neuroimaging Initiative. (2021). Validation of amyloid PET positivity thresholds in centiloids: A multisite PET study approach. *Alzheimer's Research & Therapy*, *13*(1), 99. https://doi.org/10.1186/s13195-021-00836-1

Shahid, M., Rawls, A., Ramirez, V., Ryman, S., Santini, V. E., Yang, L., Sha, S. J., Hall, J. N., Montine, T. J., Lin, A., Tian, L., Henderson, V. W., Cholerton, B., Yutsis, M., & Poston, K. L. (2023). Illusory Responses across the Lewy Body Disease Spectrum. *Annals of Neurology*, *93*(4), 702–714. https://doi.org/10.1002/ana.26574

Trelle, A. N., Carr, V. A., Guerin, S. A., Thieu, M. K., Jayakumar, M., Guo, W., Nadiadwala, A., Corso, N. K., Hunt, M. P., Litovsky, C. P., Tanner, N. J., Deutsch, G. K., Bernstein, J. D., Harrison, M. B., Khazenzon, A. M., Jiang, J., Sha, S. J., Fredericks, C. A., Rutt, B. K., …

Wagner, A. D. (2020). Hippocampal and cortical mechanisms at retrieval explain variability in episodic remembering in older adults. *eLife*, *9*, e55335. https://doi.org/10.7554/eLife.55335

Trelle, A. N., Carr, V. A., Wilson, E. N., Swarovski, M. S., Hunt, M. P., Toueg, T. N., Tran, T. T., Channappa, D., Corso, N. K., Thieu, M. K., Jayakumar, M., Nadiadwala, A., Guo, W., Tanner, N. J., Bernstein, J. D., Litovsky, C. P., Guerin, S. A., Khazenzon, A. M., Harrison, M. B., … Mormino, E. C. (2021). Association of CSF Biomarkers With Hippocampal-Dependent Memory in Preclinical Alzheimer Disease. *Neurology*, *96*(10), e1470–e1481. https://doi.org/10.1212/WNL.0000000000011477

Trelle, A. N., Young, C. B., Vossler, H., Benitez, J. R., Cody, K. A., Swarovski, M. S., Le Guen, Y., Feinstein, I., Butler III, R. R., Channappa, D., Romero, A., Park, J., Shahid-Besanti, M., Corso, N. K., Chau, K., Smith, A. N., Skylar-Scott, I., Yutsis, M. V., Fredericks, C. A., … Wilson, E. N. (Under Review). *Plasma Aβ42/Aβ40 is sensitive to early cerebral Aβ accumulation and predicts risk of cognitive decline across the Alzheimer's disease spectrum*.

Wilson, E. N., Young, C. B., Ramos Benitez, J., Swarovski, M. S., Feinstein, I., Vandijck, M., Le Guen, Y., Kasireddy, N. M., Shahid, M., Corso, N. K., Wang, Q., Kennedy, G., Trelle, A. N., Lind, B., Channappa, D., Belnap, M., Ramirez, V., Skylar-Scott, I., Younes, K., … Andreasson, K. I. (2022). Performance of a fully-automated Lumipulse plasma phospho-tau181 assay for Alzheimer's disease. *Alzheimer's Research & Therapy*, *14*(1), 172. https://doi.org/10.1186/s13195-022-01116-2

10.3. Supplementary Figures

Fig S1. Data overview.

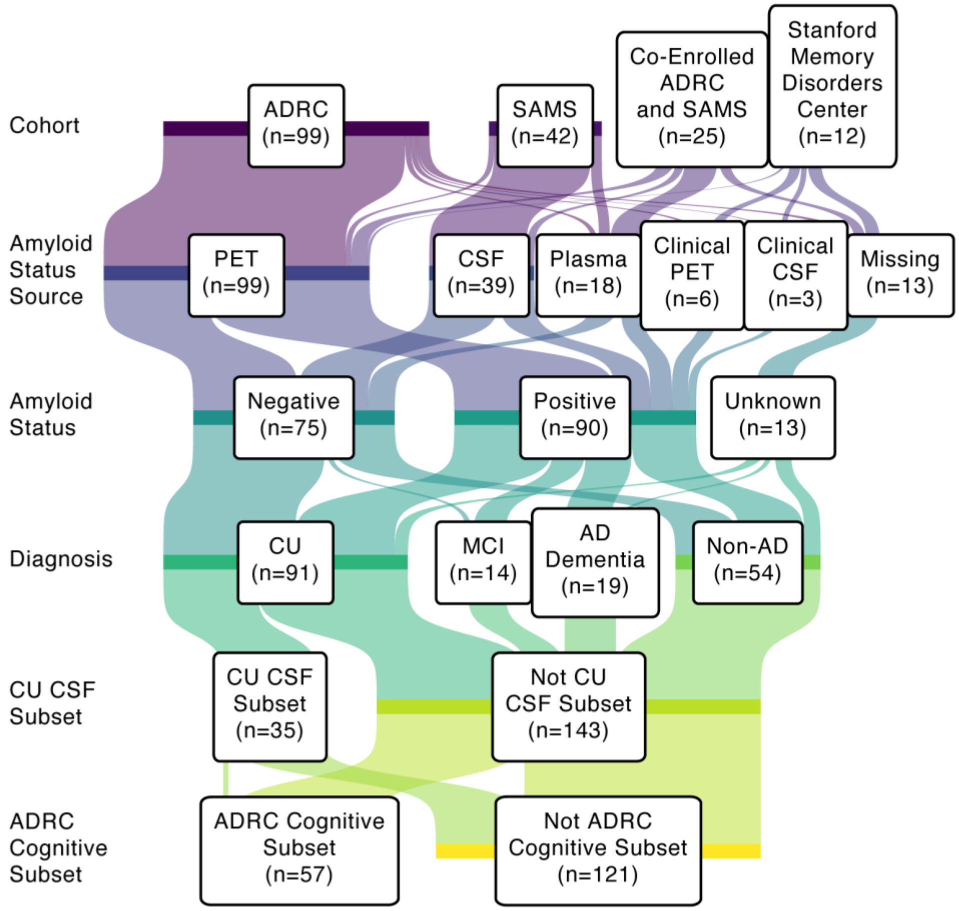

Fig S2. Comparison of SUVRs calculated from 60-90 min data to SUVRs that were interpolated to the 45-75 min scale for the 49 participants that were scanned from 60-90 min.

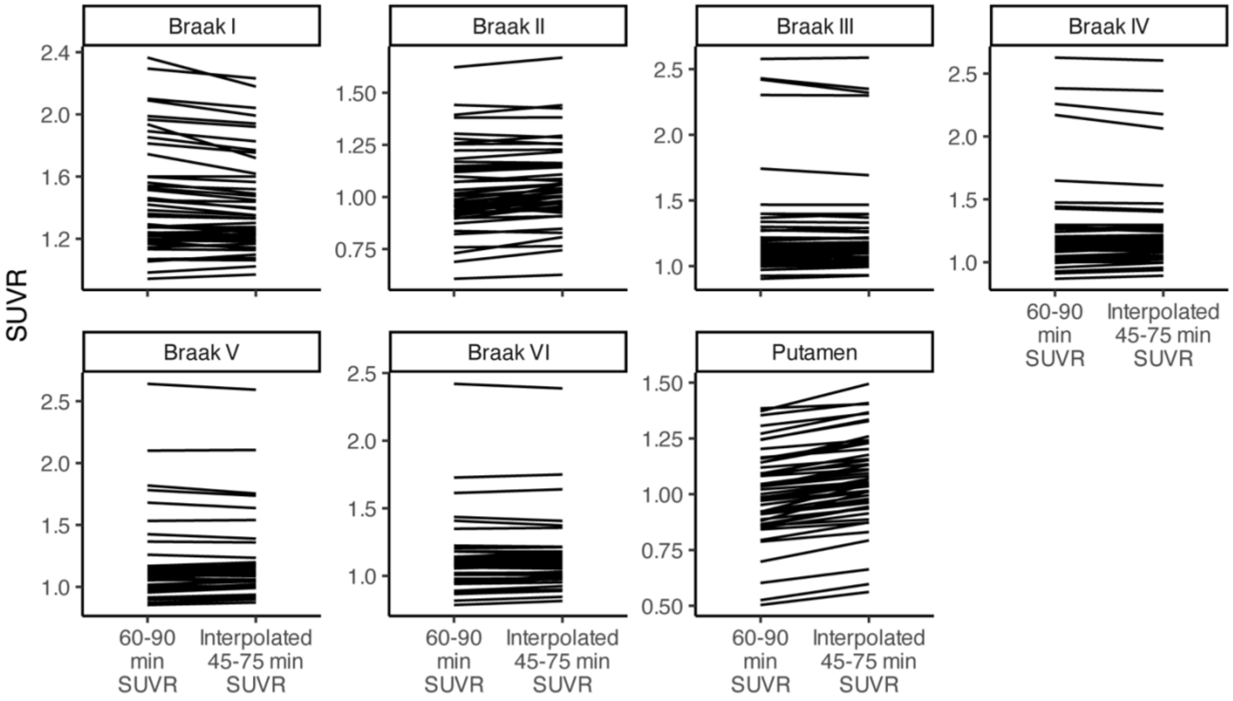

Fig S3. Effects of (A) disease severity, (B) age across diagnostic groups, and (C) age within diagnostic groups on [18F]PI-2620 SUVRs in 115 participants across the aging and AD clinical spectrum. In A, linear regression models predicting regional tau SUVR were examined ($\text{Regional SUVR}_i = B_0 + B_1{*}\text{Diagnostic Group}_i + B_2{*}\text{Age}_i + B_3{*}\text{Sex}_i + \varepsilon_i$); adjusted SUVRs account for age and sex, and significant differences between groups are shown with black lines. In B, the same linear regression models were used and adjusted SUVRs account for diagnostic group and sex; * $p < 0.05$, ** $p < 0.01$. In C, linear regression models were run separately for each diagnostic group and adjusted SUVRs account for sex.

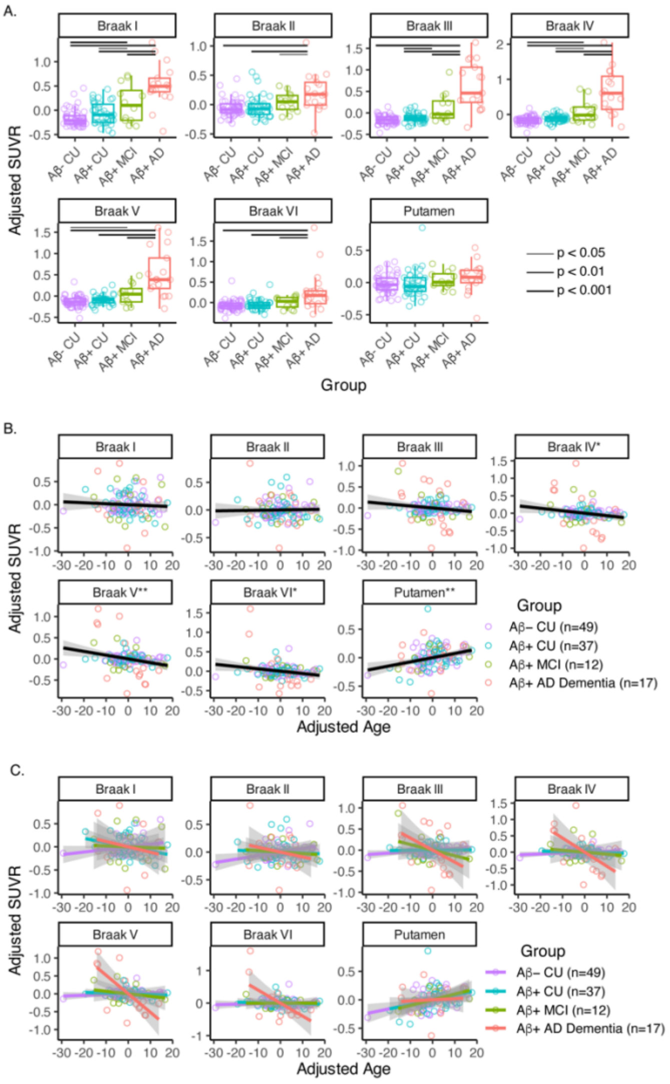

Fig S4. Comparison of SUVRs measured with 45-75 min data (n=59) and SUVRs interpolated to the 45-75 min scale (n=38) for each diagnostic group. Note that the individual groups are relatively small: Aβ- CU with 45-75 min data (n=30), Aβ- CU with interpolated 45-75 min data (n=19), Aβ+ CU with 45-75 min data (n=26), Aβ+ CU with interpolated 45-75 min data (n=11), Aβ+ MCI with 45-75 min data (n=7), Aβ+ MCI with interpolated 45-75 min data (n=5), Aβ+ AD with 45-75 min data (n=12), Aβ+ AD with interpolated 45-75 min data (n=5).

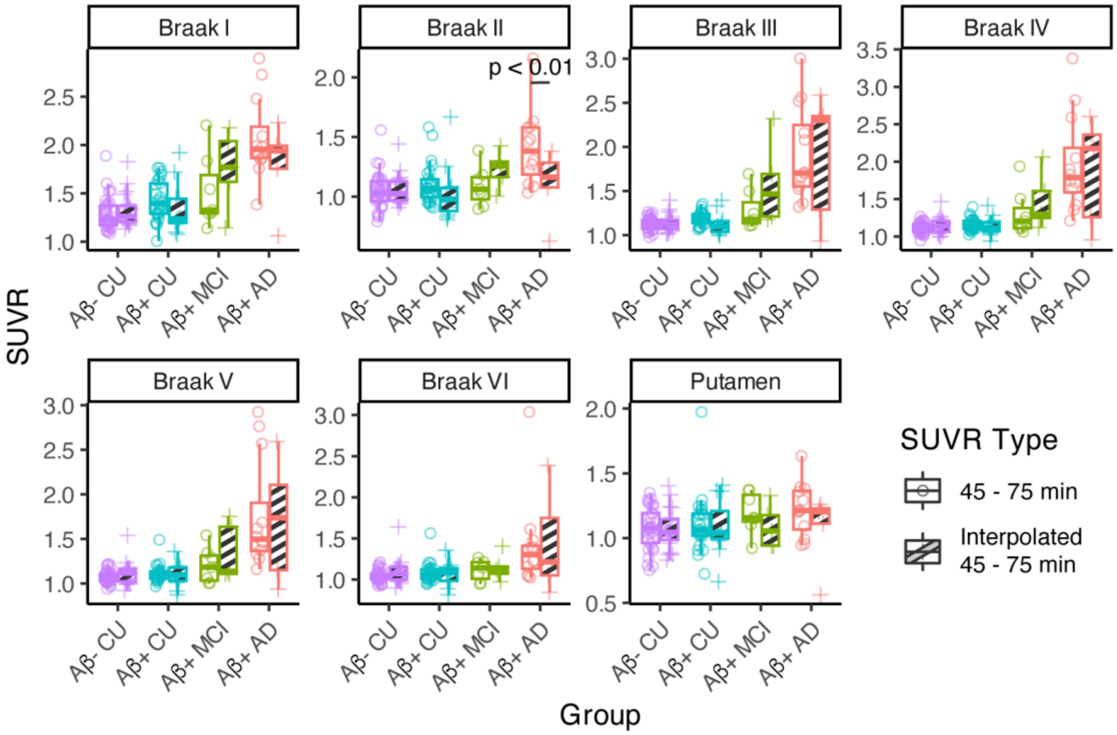

Fig S5. Associations between [18F]PI-2620 SUVR and CSF pTau-181 in 35 cognitively unimpaired (CU) participants after adjusting for amyloid status, age, and sex (linear regression model: CSF pTau-181$_i$ = $B_0 + B_1$*Amyloid Status$_i$ + B_2*Age$_i$ + B_3*Sex$_i$ + ε_i). Unstandardized betas (standard error) and p values are provided.

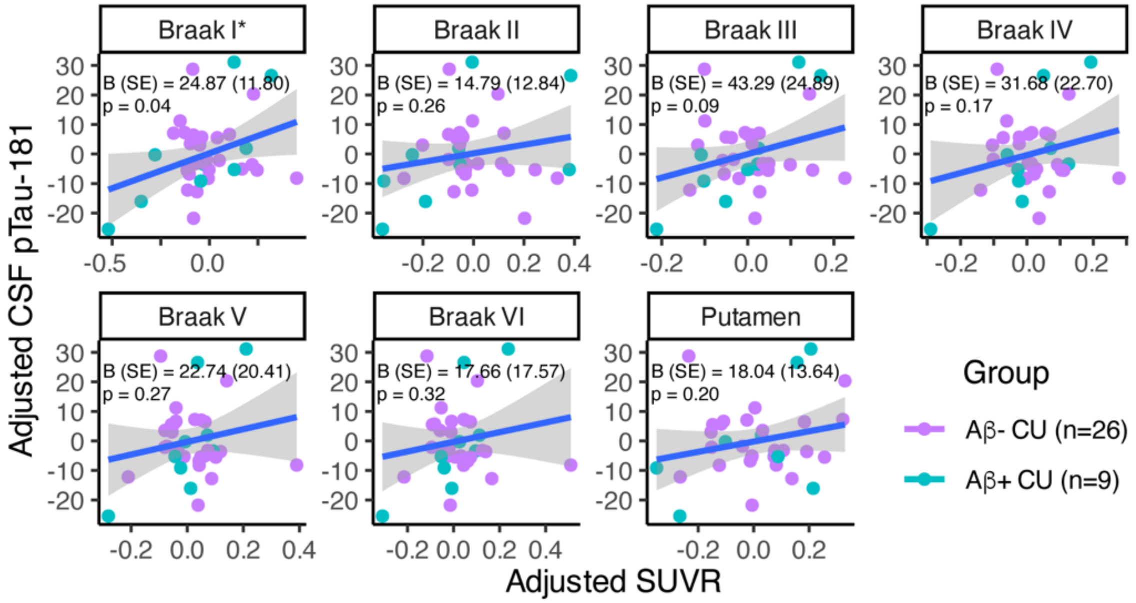

10.4. Supplementary Tables

Table S1. (A) Unstandardized and (B) standardized betas for age and sex effects on regional tau SUVR after controlling for diagnostic group (Aβ- CU, Aβ+ CU, Aβ+ MCI, Aβ+ AD Dementia) in 115 participants across the aging and AD clinical spectrum. Each row represents a separate model (Regional $Tau_i = B_0 + B_1*Age_i + B_2*Sex_i + B_3*Diagnostic\ Group_i + \varepsilon_i$).

	Age	Sex (M vs. F)
A. *Unstandardized* betas (standard error) and p values		
Braak I	-0.002 (0.003), p = 0.511	0.020 (0.052), p = 0.704
Braak II	0.001 (0.002), p = 0.816	-0.008 (0.039), p = 0.839
Braak III	-0.005 (0.003), p = 0.118	-0.057 (0.049), p = 0.251
Braak IV	**-0.007 (0.003), p = 0.035**	0.026 (0.053), p = 0.627
Braak V	**-0.009 (0.003), p = 0.004**	0.028 (0.049), p = 0.570
Braak VI	**-0.006 (0.003), p = 0.033**	-0.002 (0.045), p = 0.972
Putamen	**0.007 (0.002), p = 0.001**	0.013 (0.036), p = 0.725
B. *Standardized* betas (standard error) and p values		
Braak I	-0.048 (0.073), p = 0.511	0.028 (0.073), p = 0.704
Braak II	0.021 (0.088), p = 0.816	-0.018 (0.088), p = 0.839
Braak III	-0.102 (0.065), p = 0.118	-0.075 (0.065), p = 0.251
Braak IV	**-0.141 (0.066), p = 0.035**	0.032 (0.066), p = 0.627
Braak V	**-0.205 (0.070), p = 0.004**	0.040 (0.070), p = 0.570
Braak VI	**-0.182 (0.084), p = 0.033**	-0.003 (0.084), p = 0.972
Putamen	**0.306 (0.091), p = 0.001**	0.032 (0.091), p = 0.725

Table S2. Age and sex effects on regional [18F]PI-2620 SUVRs within each diagnostic group. Each row represents a separate model (Regional $SUVR_i = B_0 + B_1*Age_i + B_2*Sex_i + \varepsilon_i$) and *standardized* betas (standard error) and p values are provided.

	Age	Sex (M vs. F)
Within Aβ- CU (n=49)		
Braak I	**0.278 (0.137), p = 0.048**	0.249 (0.137), p = 0.075
Braak II	**0.372 (0.135), p = 0.008**	0.141 (0.135), p = 0.304
Braak III	**0.386 (0.135), p = 0.006**	0.138 (0.135), p = 0.309
Braak IV	0.259 (0.137), p = 0.065	0.266 (0.137), p = 0.059
Braak V	0.181 (0.142), p = 0.210	0.185 (0.142), p = 0.200
Braak VI	0.137 (0.143), p = 0.340	0.216 (0.143), p = 0.137
Putamen	**0.407 (0.134), p = 0.004**	0.088 (0.134), p = 0.514
Within Aβ+ CU (n=37)		
Braak I	-0.298 (0.166), p = 0.082	0.013 (0.166), p = 0.940
Braak II	-0.064 (0.174), p = 0.716	0.013 (0.174), p = 0.941
Braak III	-0.010 (0.170), p = 0.954	0.222 (0.170), p = 0.201
Braak IV	-0.142 (0.167), p = 0.403	0.271 (0.167), p = 0.115
Braak V	-0.143 (0.168), p = 0.400	0.240 (0.168), p = 0.164
Braak VI	-0.114 (0.166), p = 0.497	0.301 (0.166), p = 0.079
Putamen	0.265 (0.165), p = 0.118	0.146 (0.165), p = 0.382
Within Aβ+ MCI (n=12)		
Braak I	-0.040 (0.339), p = 0.909	-0.043 (0.339), p = 0.903
Braak II	-0.133 (0.330), p = 0.695	-0.178 (0.330), p = 0.603
Braak III	-0.345 (0.308), p = 0.292	-0.184 (0.308), p = 0.565
Braak IV	-0.190 (0.330), p = 0.579	0.174 (0.330), p = 0.610
Braak V	-0.279 (0.325), p = 0.414	0.139 (0.325), p = 0.680
Braak VI	0.017 (0.337), p = 0.960	-0.131 (0.337), p = 0.706
Putamen	0.608 (0.271), p = 0.051	-0.212 (0.271), p = 0.454
Within Aβ+ AD Dementia (n=17)		
Braak I	-0.221 (0.261), p = 0.411	-0.064 (0.261), p = 0.809
Braak II	-0.221 (0.258), p = 0.406	-0.159 (0.258), p = 0.547
Braak III	-0.413 (0.219), p = 0.080	-0.365 (0.219), p = 0.118
Braak IV	**-0.559 (0.219), p = 0.023**	-0.098 (0.219), p = 0.663
Braak V	**-0.712 (0.187), p = 0.002**	-0.042 (0.187), p = 0.827
Braak VI	**-0.610 (0.196), p = 0.008**	-0.259 (0.196), p = 0.207
Putamen	0.072 (0.263), p = 0.787	-0.196 (0.263), p = 0.470

Table S3. Regional tau effects on cognition after controlling for diagnostic group (Aβ- CU, Aβ+ CU, Aβ+ MCI, Aβ+ AD Dementia), age, sex (Male, Female), and education in 57 participants with available cognitive data. Each row represents a separate linear model ($Cognition_i = B_0 + B_1*Regional\ Tau_i + B_2*Diagnostic\ Group_i + B_3*Age_i + B_4*Sex_i + B_5*Education_i + \varepsilon_i$) and unstandardized betas (standard error) and p values are provided.

	Regional Tau	Aβ+ CU vs. Aβ- CU	Aβ+ MCI vs. Aβ- CU	Aβ+ AD Dementia vs. Aβ- CU	Age	Sex (M vs. F)	Education
Braak I							
Memory	-0.945 (0.509); p = 0.071	-0.283 (0.298); p = 0.348	**-2.002 (0.466); p < 0.001**	**-3.434 (0.479); p < 0.001**	-0.013 (0.013); p = 0.334	0.062 (0.299); p = 0.836	**0.164 (0.059); p = 0.009**
EF	**-1.034 (0.397); p = 0.013**	-0.070 (0.234); p = 0.766	**-0.795 (0.356); p = 0.032**	**-1.162 (0.369); p = 0.003**	-0.004 (0.010); p = 0.659	0.105 (0.233); p = 0.654	0.059 (0.046); p = 0.205
Language	-0.533 (0.439); p = 0.231	0.220 (0.275); p = 0.428	-0.918 (0.384); p = 0.021	**-1.371 (0.412); p = 0.002**	-0.002 (0.012); p = 0.899	0.091 (0.264); p = 0.733	-0.039 (0.056); p = 0.493
VS	0.241 (0.643); p = 0.709	0.104 (0.377); p = 0.784	0.779 (0.589); p = 0.194	-0.816 (0.606); p = 0.186	-0.022 (0.016); p = 0.189	**-0.867 (0.378); p = 0.027**	0.150 (0.075); p = 0.052
Braak II							
Memory	-0.101 (0.804); p = 0.901	-0.397 (0.307); p = 0.204	**-2.309 (0.462); p < 0.001**	**-3.952 (0.451); p < 0.001**	-0.014 (0.014); p = 0.303	0.130 (0.313); p = 0.680	**0.161 (0.063); p = 0.014**
EF	-0.401 (0.643); p = 0.536	-0.228 (0.248); p = 0.364	**-1.087 (0.366); p = 0.005**	**-1.647 (0.358); p < 0.001**	-0.005 (0.011); p = 0.631	-0.147 (0.254); p = 0.567	0.052 (0.050); p = 0.307
Language	0.439 (0.600); p = 0.468	0.147 (0.271); p = 0.590	**-1.130 (0.370); p = 0.004**	**-1.748 (0.366); p < 0.001**	0.001 (0.012); p = 0.963	0.215 (0.269); p = 0.429	-0.026 (0.057); p = 0.648
VS	**2.389 (0.898); p = 0.011**	0.248 (0.343); p = 0.475	0.571 (0.516); p = 0.276	**-1.322 (0.504); p = 0.012**	-0.028 (0.015); p = 0.079	**-0.726 (0.350); p = 0.045**	**0.186 (0.070); p = 0.012**
Braak III							
Memory	-0.837 (0.943); p = 0.380	-0.374 (0.302); p = 0.223	**-2.206 (0.466); p < 0.001**	**-3.586 (0.592); p < 0.001**	-0.012 (0.014); p = 0.409	0.150 (0.306); p = 0.626	**0.148 (0.063); p = 0.025**

EF	**-1.925 (0.692); p = 0.008**	-0.160 (0.226); p = 0.485	**-0.873 (0.341); p = 0.015**	-0.850 (0.434); p = 0.058	0.000 (0.010); p = 0.962	0.207 (0.230); p = 0.375	0.024 (0.047); p = 0.614
Language	-0.425 (0.550); p = 0.444	0.169 (0.272); p = 0.538	**-0.965 (0.393); p = 0.018**	**-1.468 (0.419); p = 0.001**	0.002 (0.012); p = 0.884	0.125 (0.264); p = 0.639	-0.043 (0.057); p = 0.458
VS	-0.156 (1.156); p = 0.893	0.135 (0.371); p = 0.717	0.882 (0.571); p = 0.130	-0.604 (0.725); p = 0.410	-0.021 (0.017); p = 0.223	**-0.883 (0.375); p = 0.024**	0.148 (0.078); p = 0.065
Braak IV							
Memory	-0.922 (0.749); p = 0.226	-0.359 (0.300); p = 0.239	**-2.132 (0.469); p < 0.001**	**-3.525 (0.536); p < 0.001**	-0.011 (0.014); p = 0.422	0.122 (0.303); p = 0.690	**0.148 (0.062); p = 0.021**
EF	**-1.820 (0.530); p = 0.001**	-0.144 (0.217); p = 0.511	**-0.757 (0.333); p = 0.029**	**-0.855 (0.379); p = 0.030**	0.000 (0.010); p = 0.965	0.133 (0.220); p = 0.551	0.028 (0.044); p = 0.525
Language	-1.079 (0.597); p = 0.077	0.206 (0.266); p = 0.441	**-0.825 (0.381); p = 0.035**	**-1.142 (0.438); p = 0.012**	0.004 (0.012); p = 0.716	0.094 (0.256); p = 0.715	-0.055 (0.056); p = 0.327
VS	1.042 (0.912); p = 0.260	0.094 (0.365); p = 0.798	0.647 (0.571); p = 0.264	-1.191 (0.652); p = 0.076	-0.026 (0.017); p = 0.131	**-0.869 (0.369); p = 0.024**	**0.167 (0.075); p = 0.032**
Braak V							
Memory	**-2.147 (0.981); p = 0.035**	-0.331 (0.289); p = 0.260	**-2.118 (0.436); p < 0.001**	**-3.292 (0.488); p < 0.001**	-0.011 (0.013); p = 0.378	0.213 (0.294); p = 0.473	**0.160 (0.058); p = 0.009**
EF	**-2.189 (0.748); p = 0.006**	-0.156 (0.224); p = 0.490	**-0.921 (0.333); p = 0.009**	**-1.052 (0.372); p = 0.008**	-0.003 (0.010); p = 0.747	0.238 (0.229); p = 0.304	0.055 (0.045); p = 0.231
Language	-0.849 (0.744); p = 0.259	0.183 (0.271); p = 0.503	**-0.932 (0.384); p = 0.019**	**-1.386 (0.413); p = 0.002**	0.002 (0.012); p = 0.876	0.149 (0.258); p = 0.567	-0.038 (0.056); p = 0.502
VS	-0.426 (1.260); p = 0.737	0.144 (0.371); p = 0.700	0.901 (0.560); p = 0.116	-0.541 (0.627); p = 0.393	-0.021 (0.017); p = 0.212	**-0.871 (0.377); p = 0.026**	0.150 (0.075); p = 0.052
Braak VI							
Memory	-1.256 (1.125); p = 0.271	-0.358 (0.301); p = 0.242	**-2.299 (0.445); p < 0.001**	**-3.765 (0.434); p < 0.001**	-0.014 (0.013); p = 0.314	0.235 (0.317); p = 0.462	**0.169 (0.061); p = 0.008**
EF	-0.350 (0.913); p = 0.703	-0.204 (0.248); p = 0.417	**-1.128 (0.360); p = 0.003**	**-1.695 (0.351); p < 0.001**	-0.006 (0.011); p = 0.584	0.198 (0.262); p = 0.456	0.060 (0.050); p = 0.237

Language	0.224 (0.872); p = 0.799	0.141 (0.274); p = 0.610	**-1.103 (0.376); p = 0.005**	**-1.682 (0.361); p < 0.001**	0.001 (0.012); p = 0.917	0.160 (0.261); p = 0.544	-0.034 (0.056); p = 0.547
VS	-1.508 (1.367); p = 0.277	0.173 (0.366); p = 0.640	0.887 (0.540); p = 0.109	-0.419 (0.528); p = 0.432	-0.020 (0.016); p = 0.215	-0.767 (0.385); p = 0.053	**0.159 (0.074); p = 0.039**
Putamen							
Memory	**-1.354 (0.657); p = 0.046**	-0.383 (0.289); p = 0.193	**-2.109 (0.441); p < 0.001**	**-3.911 (0.377); p < 0.001**	-0.003 (0.014); p = 0.848	0.132 (0.293); p = 0.655	**0.170 (0.059); p = 0.006**
EF	-0.484 (0.550); p = 0.384	-0.208 (0.245); p = 0.402	**-1.061 (0.366); p = 0.006**	**-1.732 (0.313); p < 0.001**	-0.002 (0.012); p = 0.869	0.175 (0.250); p = 0.489	0.061 (0.049); p = 0.224
Language	**-1.131 (0.559); p = 0.049**	0.179 (0.262); p = 0.498	**-0.922 (0.360); p = 0.014**	**-1.627 (0.331); p < 0.001**	0.012 (0.013); p = 0.359	0.122 (0.252); p = 0.629	-0.034 (0.054); p = 0.529
VS	0.712 (0.832); p = 0.397	0.127 (0.367); p = 0.731	0.749 (0.559); p = 0.188	-0.713 (0.478); p = 0.144	-0.028 (0.018); p = 0.128	**-0.883 (0.372); p = 0.023**	0.147 (0.074); p = 0.055

Table S4. Regional tau effects on cognition after controlling for diagnostic group (Aβ- CU, Aβ+ CU, Aβ+ MCI, Aβ+ AD Dementia), age, sex (Male, Female), and education in 57 participants with available cognitive data. Each row represents a separate linear model ($Cognition_i = B_0 + B_1*Regional\ Tau_i + B_2*Diagnostic\ Group_i + B_3*Age_i + B_4*Sex_i + B_5*Education_i + \varepsilon_i$) and *standardized* betas (standard error) and p values are provided.

	Regional Tau	Aβ+ CU vs. Aβ- CU	Aβ+ MCI vs. Aβ-CU	Aβ+ AD Dementia vs. Aβ-CU	Age	Sex (M vs. F)	Education
Braak I							
Memory	-0.182 (0.098); p = 0.071	-0.081 (0.085); p = 0.348	**-0.450 (0.105); p < 0.001**	**-0.773 (0.108); p < 0.001**	-0.070 (0.071); p = 0.334	0.018 (0.084); p = 0.836	**0.205 (0.074); p = 0.009**
EF	**-0.374 (0.144); p = 0.013**	-0.038 (0.126); p = 0.766	**-0.336 (0.151); p = 0.032**	**-0.491 (0.156); p = 0.003**	-0.046 (0.103); p = 0.659	0.056 (0.124); p = 0.654	0.139 (0.107); p = 0.205
Language	-0.172 (0.142); p = 0.231	0.105 (0.131); p = 0.428	**-0.346 (0.145); p = 0.021**	**-0.517 (0.155); p = 0.002**	0.014 (0.108); p = 0.899	0.043 (0.125); p = 0.733	-0.081 (0.117); p = 0.493
VS	0.071 (0.190); p = 0.709	0.046 (0.165); p = 0.784	0.269 (0.203); p = 0.194	-0.282 (0.209); p = 0.186	-0.184 (0.138); p = 0.189	**-0.375 (0.164); p = 0.027**	0.288 (0.143); p = 0.052
Braak II							
Memory	-0.012 (0.099); p = 0.901	-0.113 (0.088); p = 0.204	**-0.520 (0.104); p < 0.001**	**-0.889 (0.101); p < 0.001**	-0.078 (0.075); p = 0.303	0.037 (0.088); p = 0.680	**0.202 (0.079); p = 0.014**
EF	-0.093 (0.149); p = 0.536	-0.122 (0.133); p = 0.364	**-0.460 (0.155); p = 0.005**	**-0.697 (0.151); p < 0.001**	-0.054 (0.112); p = 0.631	0.078 (0.135); p = 0.567	0.122 (0.118); p = 0.307
Language	0.090 (0.124); p = 0.468	0.070 (0.130); p = 0.590	**-0.426 (0.140); p = 0.004**	**-0.659 (0.138); p < 0.001**	0.005 (0.110); p = 0.963	0.102 (0.127); p = 0.429	-0.055 (0.120); p = 0.648
VS	**0.450 (0.169); p = 0.011**	0.108 (0.150); p = 0.475	0.197 (0.178); p = 0.276	**-0.456 (0.174); p = 0.012**	-0.231 (0.128); p = 0.079	**-0.314 (0.152); p = 0.045**	**0.356 (0.135); p = 0.012**
Braak III							
Memory	-0.150 (0.169); p = 0.380	-0.107 (0.086); p = 0.223	**-0.496 (0.105); p < 0.001**	**-0.807 (0.133); p < 0.001**	-0.063 (0.075); p = 0.409	0.042 (0.086); p = 0.626	**0.185 (0.079); p = 0.025**

EF	**-0.648 (0.233); p = 0.008**	-0.086 (0.121); p = 0.485	**-0.369 (0.144); p = 0.015**	-0.360 (0.183); p = 0.058	0.005 (0.105); p = 0.962	0.110 (0.122); p = 0.375	0.056 (0.110); p = 0.614
Language	-0.128 (0.165); p = 0.444	0.081 (0.130); p = 0.538	**-0.364 (0.148); p = 0.018**	**-0.553 (0.158); p = 0.001**	0.016 (0.109); p = 0.884	0.059 (0.125); p = 0.639	-0.090 (0.120); p = 0.458
VS	-0.043 (0.317); p = 0.893	0.059 (0.162); p = 0.717	0.304 (0.197); p = 0.130	-0.208 (0.250); p = 0.410	-0.176 (0.142); p = 0.223	**-0.382 (0.163); p = 0.024**	0.283 (0.149); p = 0.065
Braak IV							
Memory	-0.139 (0.113); p = 0.226	-0.102 (0.086); p = 0.239	**-0.480 (0.106); p < 0.001**	**-0.793 (0.121); p < 0.001**	-0.060 (0.074); p = 0.422	0.034 (0.086); p = 0.690	**0.185 (0.077); p = 0.021**
EF	**-0.515 (0.150); p = 0.001**	-0.077 (0.116); p = 0.511	**-0.320 (0.141); p = 0.029**	**-0.362 (0.160); p = 0.030**	0.004 (0.099); p = 0.965	0.070 (0.117); p = 0.551	0.067 (0.104); p = 0.525
Language	-0.272 (0.151); p = 0.077	0.099 (0.127); p = 0.441	**-0.311 (0.144); p = 0.035**	**-0.431 (0.165); p = 0.012**	0.039 (0.107); p = 0.716	0.044 (0.121); p = 0.715	-0.116 (0.117); p = 0.327
VS	0.241 (0.211); p = 0.260	0.041 (0.160); p = 0.798	0.223 (0.197); p = 0.264	-0.411 (0.225); p = 0.076	-0.214 (0.138); p = 0.131	**-0.376 (0.160); p = 0.024**	**0.320 (0.144); p = 0.032**
Braak V							
Memory	**-0.236 (0.108); p = 0.035**	-0.094 (0.082); p = 0.260	**-0.477 (0.098); p < 0.001**	**-0.741 (0.110); p < 0.001**	-0.063 (0.070); p = 0.378	0.060 (0.083); p = 0.473	**0.200 (0.073); p = 0.009**
EF	**-0.453 (0.155); p = 0.006**	-0.084 (0.120); p = 0.490	**-0.390 (0.141); p = 0.009**	**-0.445 (0.157); p = 0.008**	-0.033 (0.101); p = 0.747	0.126 (0.121); p = 0.304	0.128 (0.105); p = 0.231
Language	-0.157 (0.137); p = 0.259	0.087 (0.129); p = 0.503	**-0.351 (0.145); p = 0.019**	**-0.523 (0.156); p = 0.002**	0.017 (0.108); p = 0.876	0.070 (0.122); p = 0.567	-0.079 (0.117); p = 0.502
VS	-0.072 (0.213); p = 0.737	0.063 (0.162); p = 0.700	0.311 (0.193); p = 0.116	-0.187 (0.216); p = 0.393	-0.175 (0.138); p = 0.212	**-0.377 (0.163); p = 0.026**	0.287 (0.144); p = 0.052
Braak VI							
Memory	-0.102 (0.091); p = 0.271	-0.102 (0.086); p = 0.242	**-0.517 (0.100); p < 0.001**	**-0.847 (0.098); p < 0.001**	-0.074 (0.073); p = 0.314	0.066 (0.089); p = 0.462	**0.212 (0.076); p = 0.008**
EF	-0.054 (0.139); p = 0.703	-0.109 (0.133); p = 0.417	**-0.477 (0.152); p = 0.003**	**-0.717 (0.149); p < 0.001**	-0.062 (0.112); p = 0.584	0.105 (0.139); p = 0.456	0.141 (0.117); p = 0.237

Language	0.030 (0.119); p = 0.799	0.067 (0.131); p = 0.610	**-0.416 (0.142); p = 0.005**	**-0.634 (0.136); p < 0.001**	0.012 (0.110); p = 0.917	0.076 (0.124); p = 0.544	-0.072 (0.118); p = 0.547
VS	-0.188 (0.170); p = 0.277	0.076 (0.160); p = 0.640	0.306 (0.186); p = 0.109	-0.145 (0.182); p = 0.432	-0.171 (0.136); p = 0.215	-0.332 (0.166); p = 0.053	**0.304 (0.142); p = 0.039**
Putamen							
Memory	**-0.165 (0.080); p = 0.046**	-0.109 (0.083); p = 0.193	**-0.475 (0.099); p < 0.001**	**-0.880 (0.085); p < 0.001**	-0.015 (0.077); p = 0.848	0.037 (0.083); p = 0.655	**0.212 (0.074); p = 0.006**
EF	-0.111 (0.126); p = 0.384	-0.112 (0.132); p = 0.402	**-0.449 (0.155); p = 0.006**	**-0.733 (0.132); p < 0.001**	-0.020 (0.122); p = 0.869	0.093 (0.133); p = 0.489	0.143 (0.116); p = 0.224
Language	**-0.231 (0.114); p = 0.049**	0.086 (0.125); p = 0.498	**-0.348 (0.136); p = 0.014**	**-0.613 (0.125); p < 0.001**	0.107 (0.115); p = 0.359	0.058 (0.119); p = 0.629	-0.072 (0.114); p = 0.529
VS	0.133 (0.155); p = 0.397	0.056 (0.160); p = 0.731	0.258 (0.193); p = 0.188	-0.246 (0.165); p = 0.144	-0.232 (0.149); p = 0.128	**-0.382 (0.161); p = 0.023**	0.282 (0.143); p = 0.055
